# Platelet proteome for predictive diagnosis and differentiation of sepsis and septic shock in pediatric patients

**DOI:** 10.7717/peerj.20844

**Published:** 2026-03-06

**Authors:** Yiqiu Cao, Keran Chen, Chaofei Chen, Mengjie Qiu, Feiyan Chen, Lei Zhao, Fan Li, Jian Luo, Wai To Tang, Yiyun Wang, Meiling Su

**Affiliations:** 1 Yangzhi Rehabilitation Hospital (Sunshine Rehabilitation Centre), School of Medicine, Tongji University, Shanghai, China; 2 Institute of Pediatrics, Guangzhou Women and Children’s Medical Centre, Guangzhou Medical University, Guangzhou, China; 3 School of Nursing and Health Studies, Hong Kong Metropolitan University, Ho Man Tin, Kowloon, Hongkong, China

**Keywords:** DEPs, Platelets, Proteomics, Sepsis severity

## Abstract

**Objectives:**

Platelet hyperreactivity and thrombocytopenia are strongly correlated with elevated mortality rates in sepsis, particularly in cases of septic shock. This study aimed to predict pediatric sepsis and distinguish it from septic shock by profiling the platelet proteome.

**Methods:**

We conducted a comparative proteomic analysis of platelet protein expression in five individuals with sepsis, five individuals with septic shock and five healthy subjects, utilizing mass spectrometry (DIA-MS).

**Results:**

Our proteomic analysis identified that 316 and 83 differentially expressed proteins (DEPs) in sepsis and septic shock groups, respectively, each compared to the control group. Kyoto Encyclopedia of Genes and Genomes (KEGG) analysis unveiled that the DEPs in patients with clinical spectrum of sepsis severity were associated with molecular functions. Comparative Gene Ontology (GO) analysis of DEPs demonstrated distinct spatial enrichment: while ‘extracellular region’ was the top altered term in sepsis, septic shock patients displayed significant enrichment in ‘extracellular region’ and ‘extracellular space’. KEGG pathway analysis identified enrichment of DEPs in pathways related to ‘Lysosome’. Protein–protein interaction analysis identified that a set of ribosomal proteins S27a (40S), L9 (60S), P0, SA, and S3a could serve as potential discriminators between sepsis and healthy subjects. Crucially, Vesicle-associated membrane protein (VAMP) 8, (VAMP)2, Syntaxin-16 and Synaptosomal-associated protein 23 were identified as key candidates with the potential to distinguish sepsis from septic shock.

**Conclusions:**

These observed proteomic changes could inform the future biomarker identification for sepsis severity stratification. Importantly, these are preliminary findings from a small sample with limited functional assessments, and their clinical utility requires confirmation in independent, larger cohorts.

## Introduction

Pediatric sepsis, characterized by dysregulation of the immune system, abnormal platelet function, an excessive inflammatory response, and systemic multi-organ dysfunction, is acknowledged globally as a significant public health issue, associated with high morbidity and mortality (Rudd et al., 2020). There are estimated 22 cases of childhood sepsis per 100,000 people per year and 2,202 cases of neonatal sepsis per 100,000 live births, resulting in approximately 1.2 million cases of childhood sepsis annually (Fleischmann-Struzek et al., 2018). Septic shock in pediatrics continues to be a significant cause of admissions and deaths in the Pediatric Intensive Care Unit, imposing a substantial burden on healthcare costs (Ruth et al., 2014; Schlapbach et al., 2015). Sepsis-induced alterations in platelet counts (*e.g.*, thrombocytopenia) and function (*e.g.*, coagulation, immunothrombosis, immune regulation and antimicrobial defense) are associated with adverse outcomes (Cheng et al., 2024; Clark et al., 2007; Claushuis et al., 2016; Si et al., 2025; Wang et al., 2025b; Xin et al., 2022). Thrombocytopenia serves as a critical predictor of adverse outcomes in sepsis (Giustozzi et al., 2021). However, whether the distinct platelet proteins can serve as reliable diagnostic and differential biomarkers of severity degrees of sepsis have not been clearly defined.

Platelets, which are anucleate fragments derived from bone marrow megakaryocytes, contain a complex transcriptional repertoire that includes mRNAs, pre-mRNAs, and microRNAs (Rowley, Schwertz & Weyrich, 2012; Shen et al., 2021; Simon et al., 2014). A previous study has indicated that the platelet transcriptional and translational landscape underwent changes in human and murine sepsis (Middleton et al., 2019) Our hypothesis that sepsis alters the platelet proteome was driven by previous reports of specific protein changes (Hu, Li & Wang, 2012), coupled with established evidence of profound transcriptomic and translational reprogramming in septic platelets (Middleton et al., 2019), which originates from sepsis-induced alterations in megakaryocyte transcription (Ajanel & Middleton, 2023). Consequently, our study aimed to systematically characterize these predicted proteomic alterations in a pediatric cohort to identify severity-associated signatures. Most platelet RNA and protein expression results from megakaryocytes, but platelets are also capable of carrying out mRNA splicing and translating into proteins (Bray et al., 2013). In our recent study, we documented distinct expression profiles of platelet-associated proteins that are implicated in various biological processes, including apoptosis, autophagy, and pyroptosis, upon comparing patients with severe sepsis to HS (Su et al., 2022). A Phoenix Sepsis Score of 2 or higher indicates organ dysfunction (respiratory, cardiovascular, coagulation, and/or neurological) in children with suspected or confirmed infection. The diagnostic criteria for pediatric septic shock require at least 1 point in the cardiovascular component of the Phoenix Sepsis Score, defined by age-specific severe hypotension, blood lactate > five mmol/L, or the administration of vasoactive medication therapy (Schlapbach et al., 2024). Nevertheless, the specific expression patterns and functionalities of the platelet proteome in patients with sepsis and septic shock remain to be elucidated.

This study employed DIA-MS proteomics and bioinformatics to explore the correlation among proteomics of platelets, severity of sepsis, and prognosis of the patients. We observed distinct proteomic profiles in platelets derived from patients with varying sepsis severity. Potential biomarkers for predictive diagnosis and differentiation of sepsis severity included VAMP2, SNAP23, STX16, and VAMP8. The present study suggested that platelet proteins might serve as predictive biomarkers for sepsis severity and clinical outcomes, thereby underscoring the imperative for further investigation to delineate their mechanistic roles and assess their feasibility as therapeutic targets.

## Materials and Methods

### Patient recruitment and isolation of human platelets

We recruited 10 pediatric sepsis patients (aged < 18 years) and five healthy subjects (HS) from Guangzhou Women and Pediatrics’s Medical Center, Guangzhou Medical University, China. Patients with congenital immune deficiencies, apparent genetic metabolic diseases, severe cardiovascular diseases, hemophagocytic syndrome, and severe malformations were excluded from this study. The patients we collected were not on any antiplatelet or anticoagulant medications. The administered medications were limited to standard beta-lactam antibiotics (*e.g.*, penicillins and cephalosporins). Our study was approved by the Institutional Review Board of Guangzhou Women and Pediatrics’s Medical Center. Informed consent was obtained from each subject (Human Investigation Committee No. 2022-492B00 and the human studies conformed to the principles set out in the World Medical Association Declaration of Helsinki. Written informed consent was obtained. As previously described in our study (Su et al., 2022), venous blood samples were taken from patients with sepsis and septic shock when they were enrolled on the second day following hospital admission. Venous blood samples (2–3 mL) were drawn from HS and patients with different severity degrees of sepsis using blood collection tubes containing 3.8% trisodium citrate. Then, platelet suspensions were treated with one µM prostaglandin E1 (Sigma, catalog no. 745-65-3) and centrifuged at 640× g for 5 min. After discarding the supernatant, the platelet pellet was washed and resuspended in three mL Hank’s Balanced Salt Solution (HBSS; catalog no. 14025092, Gibco, Waltham, MA, USA).

### Definition of sepsis

(I) A child with suspected or confirmed infection is diagnosed with sepsis if the Phoenix Sepsis Score is ≥ 2 points. This scoring system evaluates dysfunction across four organ systems (cardiovascular, respiratory, coagulation, and neurological), quantifying the severity of impairment through specific criteria.

① Cardiovascular system: Scored based on age-specific mean arterial pressure (MAP), lactate levels, and vasoactive drug use. ② Respiratory System: Assessed *via* the ratio of arterial oxygen partial pressure to inspired oxygen concentration (PaO_2_/FiO_2_) or pulse oximetric saturation to inspired oxygen concentration (SpO_2_/FiO_2_). A score of 1 point is assigned if SpO_2_/FiO_2_ < 220 with respiratory support. ③ Coagulation System: Scores 1 point for any of the following: platelet count <100 × 10^3^/μL, international normalized ratio (INR) >1.3, D-dimer > 2 mg/L FEU, or fibrinogen < 100 mg/dL. ④ Neurological System: Glasgow Coma Scale (GCS) ≤ 10 scores 1 point, while bilateral fixed pupils score 2 points.

(II) Septic shock was defined as sepsis with a cardiovascular component score of ≥ 1 point.

In the context of sepsis, septic shock is diagnosed if the cardiovascular score is ≥ 1 point (*e.g.*, vasoactive drug use, lactate ≥ five mmol/L, or age-specific hypotension) (Schlapbach et al., 2024).

### Protein extraction

First, the samples were prepared by adding SDS-free L3 lysis buffer (7 M urea, 2 M thiourea, 20 mM Tris-HCl, pH 8.0), 2 mM EDTA, and a 1 × protease inhibitor cocktail. To control for the direct quantitative impact of platelet count on our measurements, we standardized all samples by loading equal total protein amounts for mass spectrometry analysis. The samples were placed on ice for 5 min, followed by the addition of Dithiothreitol (DTT) to achieve a final concentration of 10 mM. The lysates were then subjected to mechanical disruption (*e.g.*, by sonication or homogenization) for 2 min, followed by centrifugation at 25,000 × g for 15 min at 4 °C. The resulting supernatant was collected as the protein extract. To complete the procedure, adjusted the final concentration of DTT to 10 mM and incubated the extract in a 56 °C water bath for 1 h. Allowed the solution to cool to room temperature, and then added 55 mM iodoacetamide (IAM). Incubated the mixture in a dark environment at room temperature for 45 min. Finally, proceeded with quantitative electrophoresis analysis (Ma et al., 2020; Zhu et al., 2020).

### Protein extraction quality control

Bradford quantification: The standard protein (0.2 μg/μL bovine serum albumin (BSA)) was added sequentially to positions A1 to A10 of the 96-well microtiter plates, followed by the addition of purified water sequentially, and then 180 μL of Coomassie Brilliant Blue G-250 Quantitative Working Solution was added to each well. The optical density (OD)595 was measured using a microplate reader, and a linear standard curve was generated based on the OD_595_ *versus* protein concentration. The protein solution was diluted and assayed, and 180 microliters (μL) of quantitative working solution was added to 20 μL of the diluted protein solution. The OD was then measured. We calculated the protein concentration of the sample based on the standard curve and sample OD_595_.

SDS-PAGE: For each sample, 10 µg of protein solution was added to 4 ×loading buffer which was composed of Tris–HCl (pH 6.8, 1.0 M), SDS, and DTT and then thoroughly mixed. The mixture was then heated at 95 °C for 5 mins and subsequently centrifuged at 25,000 × g for 5 mins. The obtained supernatant was aliquoted into the wells of a 12% Tris-glycine SDS-polyacrylamide gel electrophoresis (SDS-PAGE) gel. The gel was then subjected to electrophoresis at constant voltage at 80 V for an initial period of 30 mins, followed by a subsequent electrophoresis step at 120 V for a duration of 120 mins. After completion of electrophoresis, the gel was placed in a rapid dye-off instrument for 10 mins and subsequently removed for scanning.

### Protein enzymatic hydrolysis

Each sample was prepared using a protein solution containing 100 µg, and 2.5 µg of trypsin enzyme was added, corresponding to a protein-to-enzyme ratio of 40:1. Digestion was performed at 37 °C for 4 h. Enzymatic peptides were desalted using a Strata X column and then subjected to vacuum drying.

### High-pH reversed-phase separation

Equal amounts of peptides were aliquoted from each sample and then pooled. The mixture was diluted with mobile phase A (5% acetonitrile (ACN), pH 9.8) and injected into a Shimadzu LC-20AB system for separation. The separation process involved a flow rate gradient of one mL/min. Initially, the mobile phase consisted of 10% mobile phase B (95% ACN, pH 9.8) for 10 mins. This was followed by a linear increase in mobile phase B from 5% to 35% over 40 mins, then from 35% to 95% for 1 min. The mobile phase B was then maintained for 3 min before returning to an equilibrium state with 5% mobile phase B for 10 min. During the separation, elution peaks were monitored at 214 nm and one component was collected every minute. These collected samples were combined. In total, 10 fractions were obtained by combining the plots of the elution peaks and subsequently freeze-dried (Fang et al., 2023; Ma et al., 2020; Zhu et al., 2020).

### Data-dependent and data-independent acquisition analyses

The dried peptide samples were reconstituted in mobile phase A (2% ACN, 0.1% FA) and then centrifuged at 20,000× g for 10 mins. The supernatant was collected as the sample. The separation was carried out using Thermo UltiMate 3000 UHPLC liquid chromatograph. Firstly, the sample was enriched and desalted on a trap column, and then it was separated on a self-packed C18 column (150 µm internal diameter, 1.8 µm particle size) using a gradient at a flow rate of 500 nL/min. The gradient was as follows: 5% B (98% ACN, 0.1% FA) for 0–5 mins; a linear increase from 5% to 25% B over 5–120 mins; an increase from 25% to 35% B over 120–125 mins; and an increase from 35% to 90% B over 125–135 mins. The nanoliter liquid phase separation end was directly connected to the mass spectrometer and detected.

### DDA library construction detection and DIA mass spectrometry detection

The peptides separated by liquid phase chromatography were ionized by a nanoESI source and then analyzed by a tandem mass spectrometer Orbitrap Exploris 480 (Thermo Fisher Scientific, Waltham, MA, USA) both for data-dependent acquisition (DDA) mode detection and data-independent acquisition (DIA) mode detection. The main parameters were set: ion source voltage was set to 1.9 kV, MS1 mass spectrometer scanning range was 350∼1,650 m/z; resolution was set to 120,000; maximum injection time (MIT) 90 ms; MS/MS collision type HCD, collision energy NCE 30%; MS/MS resolution 30,000, MIT was auto mode, dynamic exclusion duration 120 s. The start m/z for MS/MS was fixed to auto mode. Precursor for MS/MS scan met the following criteria: charge range 2+ to 6+, top 30 precursors with intensity over 2E4. AGC was: MS 300%, MS/MS 100%.

### Functional and pathway enrichment analysis

To enhance our understanding of the functions and roles of DEPs across various severity of sepsis severity, we firstly performed GO and KEGG pathway enrichment analyses utilizing the online platform (https://biosys.bgi.com). Next, we identified the top five GO and KEGG pathways and used GraphPad Prism 8.0 to create graphs.

### Relative protein expression

We collected all DEPs expression data including fold-change and *P* value between different severity of sepsis and HS from the online platform (https://biosys.bgi.com). These data were compiled into a table, which included nine key proteins of interest. Then, we used GraphPad Prism 8.0 to generate the graphs,

### Data analysis

DDA data were processed to construct a spectral library and DIA data were searched and analyzed using FragPipe software (version 20.0). The enzyme was set to trypsin, and the maximum missed cleavages were two. The fixed modification site was carbamidomethyl (C), and the variable modification were oxidation (M), acetylation (protein N-term), glutamine to pyro-glutamic acid conversion (N-term Q), and deamidation (NQ). The minimum peptide length was 7 amino acids. We employed iRT peptides for retention time calibration. False positives for proteins and spectra were controlled to be less than 1% FDR. Peak area intensity values were extracted and protein quantification values were calculated. Then, the fold change of proteins in each comparison group was calculated separately according to the set comparison groups and tested for significance using Welch’s t-test. Further, screening was performed based on the |fold change|≥ 1.5 and *P* value < 0.05 for DEPs.

### Flow cytometric analysis

The platelet preparation purity was assessed *via* flow cytometry with PE-conjugated anti-human CD62P antibody (BD Biosciences, Franklin Lakes, NJ, USA; dilution 1:200; catalog no. 550561). Using a BD FACSCanto flow cytometer, 20,000 platelet events were acquired, and subsequent data analysis was performed with FlowJo software (version 10).

### Western blotting

Protein extraction was performed by lysing cells in RIPA buffer supplemented with protease inhibitor cocktail (Millipore, Burlington, MA, USA). Following separation *via* SDS-PAGE, proteins were electrophoretically transferred onto PVDF membranes. After blocking nonspecific binding sites with 5% skim milk (2 h, room temperature), membranes were probed overnight at 4 °C with primary antibody: VAMP2 antibody (FineTest; 1:500; FNab09359). Subsequent incubation with species-matched HRP-conjugated secondary antibodies (1:2,000) was followed by chemiluminescent detection using ECL reagent (Millipore, Burlington, MA, USA). Band intensity quantification was conducted with Image Lab software (Bio-Rad, Hercules, CA, USA).

### Statistical analysis

The statistical analyses were performed using GraphPad Prism 8.0 software. Data were expressed as mean ± SD unless otherwise stated. For variables with overall *P* values greater than 0.05, we did not perform group comparisons. For normally distributed data, comparisons between two groups were analyzed using unpaired Students’ *t*-test and comparisons among more than two groups were analyzed using one-way analysis of variance (ANOVA) with Tukey’s multiple comparisons. For data that were not distributed normally, comparisons among more than two groups were performed using Kruskal-Wallis test with Dunn’s multiple comparison test. Pearson’s correlation test was used to analyze the linear relationship between two variables.

### Data availability

The mass spectrometry proteomics data have been deposited to the ProteomeXchange Consortium *via* the iProX partner repository with the dataset identifier PXD053408/IPX0009129002. Source data are provided with this article.

## Results

### Characterization of the study cohort and platelet DEPs identification in sepsis patients

In our study, a control group comprising five patients was designated as the HS. We enrolled ten subjects who were diagnosed with sepsis and categorized them into two groups: five with sepsis and five with septic shock. Table 1 primarily presents the demographic information and baseline laboratory test results between septic patients and healthy subjects (Fig. S1). Patients diagnosed with septic shock with platelet counts below 50 × 10^9^/L, exhibited reduced platelet levels. This association suggests that platelet counts may serve as an indicator of sepsis severity, disease progression, and clinical outcomes.

**Table 1:** Demographic data for the cohort of septic patients and healthy subjects.

Characteristics	Healthy subjects (*n* = 5)	Sepsis (*n* = 5)	Shock (*n* = 5)	Healthy subjects *vs* sepsis	Healthy subjects *vs* shock	Sepsis *vs* shock
Age <18 years, n (%)	5 (100)	5 (100)	5 (100)	–	–	–
Sex (male), n (%)	2 (40)	3 (60)	1 (20)	–	–	–
PLT (10^9^/L)	305.60 ± 38.60	237.80 ± 111.03	46.00 ± 54.59	NS	0.0089	NS
WBC (10^9^/L)	10.46 ± 1.10	14.72 ± 12.75	15.92 ± 14.94	NS	NS	NS
Neutrophil ratio (%)	37.80 ± 16.48	64.80 ± 13.61	37.80 ± 18.97	NS	NS	NS
Lymphocyte ratio (%)	43.40 ± 22.12	17.80 ± 12.40	32.20 ± 22.22	NS	NS	NS
RBC (10^12^/L)	5.31 ± 0.47	3.95 ± 0.74	3.41 ± 0.34	0.0051	0.0003	NS
HGB (g/L)	169.20 ± 31.45	106.00 ± 24.70	99.40 ± 12.62	0.0037	0.0018	NS
MPV (fL)	10.14 ± 0.66	10.62 ± 0.74	11.36 ± 0.85	NS	NS	NS
PDW (fL)	11.12 ± 1.44	12.92 ± 2.68	12.06 ± 1.52	NS	NS	NS
P-LCR (%)	25.16 ± 5.09	29.98 ± 6.59	35.20 ± 7.07	NS	NS	NS

**Notes.**

Data were expressed as mean ± SD unless otherwise stated. For variables with overall *p* values greater than 0.05, we did not perform group comparisons. One-way ANOVA and Tukey’s multiple comparisons test for WBC, Neutrophil ratio, Lymphocyte ratio, RBC, HGB, MPV, PDW and P-LCR. Kruskall-Wallis test and Dunn’s multiple comparisons test for PLT.

Abbreviations WBC white blood cell RBC red blood cell HGB hemoglobin MPV mean platelet volume PDW platelet distribution width P-LCR platelet-larger cell ratio NS non-significant differences

**Figure 1:**
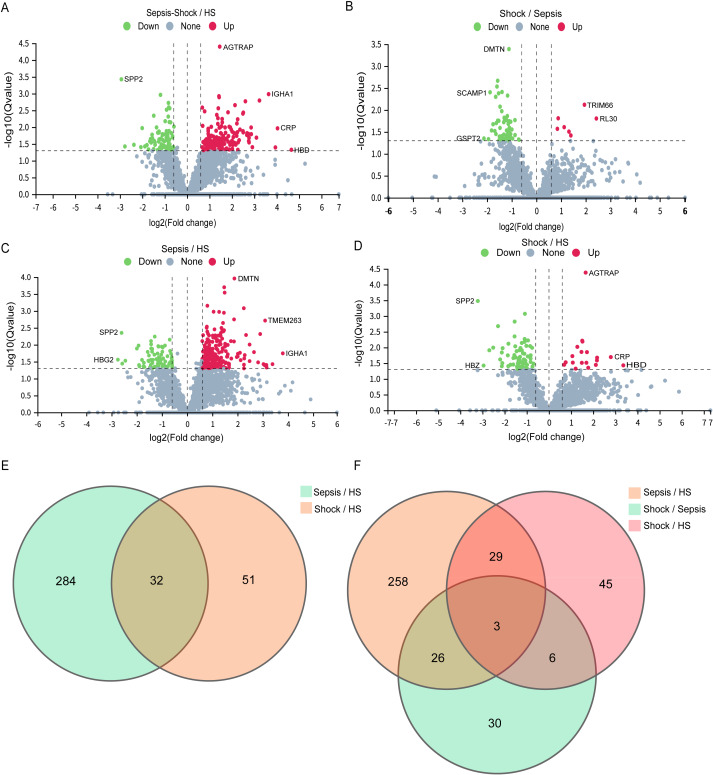
Identification of DEPs in platelets of sepsis patients. (A) DEPs between patients with sepsis and shock with HS. The *x*-axis represents protein difference (log2-transformed fold changes), and the *y*-axis represents the corresponding *p*-value (log10 converted). Up-regulated proteins are represented by red dots, down-regulated proteins by green dots, and proteins with no significant change by grey dots. (B) DEPs among patients with sepsis compared to shock. Each circle in the figure represents the set of DEPs for a particular comparison group. The overlapping regions indicate the proteins that are common to multiple sets, while the unstacked portion represents the DEPs exclusive to that specific comparison group. (C) DEPs among patients with sepsis compared to HS. (D) DEPs overlap relationships among patients with s hock compared to HS. (E) DEPs overlap relationships between patients with sepsis, shock with HS. Each circle in the figure represents the set of DEPs for a particular comparison group. The overlapping regions indicate the proteins that are common to multiple sets, while the unstacked portion represents the DEPs exclusive to that specific comparison group. (F) DEPs overlap relationships between patients with sepsis, shock with HS and sepsis patients compared to shock.

To further identify the changes in the platelets of sepsis patients, we performed high-throughput proteomic analysis in purified platelets with DIA-MS from patients with sepsis, septic shock, and HS. As a result, a total of 236 proteins were identified in the platelets of sepsis, septic shock, and HS (Fig. 1A). Among these DEPs, 156 were up-regulated in expression and 80 were down-regulated (Fig. S2B). More detailed information regarding the proteins identified by DDA can be found in Table S1. Subsequently, we conducted the mass spectrometry data collection with the DIA. For significant DEPs, we adopted screening criteria of |fold change|≥ 1.5 and false discovery rate <0.05. We identified a total of 316 DEPs in patients with sepsis compared to HS. Among these DEPs, 241 were up-regulated in expression and 75 were down-regulated (Fig. 1C and Fig. S2A). Similarly, compared to HS, 83 proteins showed differential expression in patients with septic shock. Out of these DEPs, 19 were up-regulated in expression and 64 were down-regulated (Fig. 1D and Fig. S2A).

In addition, we also compared DEP expression among patients with different severity of sepsis. Our analysis revealed that 65 proteins showed differential expression in patients with septic shock compared to those with sepsis. Out of these DEPs, seven were up-regulated in expression and 58 were down-regulated (Fig. 1B and Fig. S2A). Taken together, compared to HS, DEPs were significantly up-regulated in patients with sepsis, but markedly down-regulated in those with septic shock.

**Figure 2:**
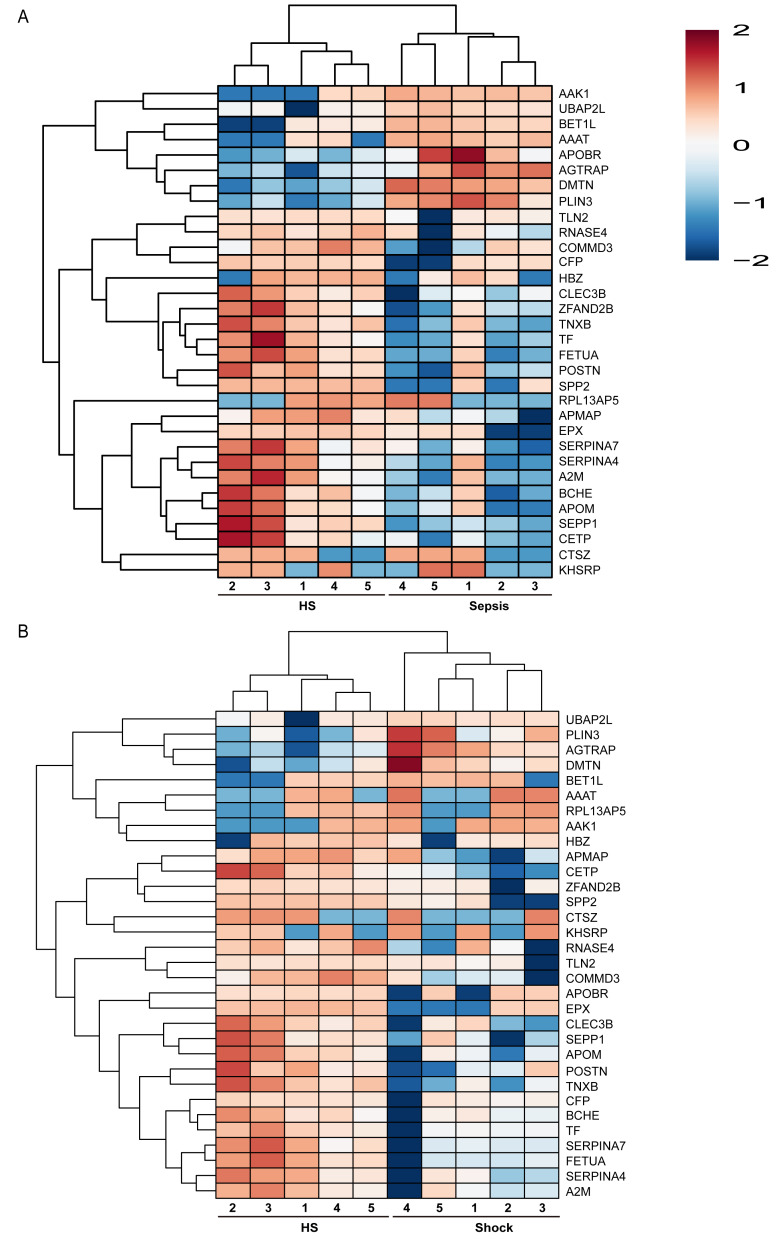
Cluster analysis chart of the 32 DEPs between patients with different severity of sepsis and HS. Cluster analysis chart of the 32 DEPs between patients with sepsis and HS (A); between patients with septic shock and HS (B). Higher red and blue intensities indicate higher degree of up- and down-regulated respectively.

To elucidate the significant proteomic distinctions between sepsis and HS, we pinpointed the 32 shared prevalently expressed proteins within the platelets of patients exhibiting sepsis and septic shock, as depicted in Fig. 1E. Additionally, comparisons (sepsis *vs.* HS and sepsis *vs.* septic shock) revealed 3 proteins with significant differences across all contrasts (Fig. 1F). From these, 32 DEPs with the representative expression differences were selected for visualization through a cluster analysis plot (Fig. 2A and 2B), illustrating the expression differences between the groups. Further information regarding the functional classification and UniProt details of the 32 shared DEPs can be found in Table S2. Our findings hinted at the possibility that different levels of sepsis might lead to changes in the platelet proteome, which could mirror the varying impacts of the disease.

**Figure 3:**
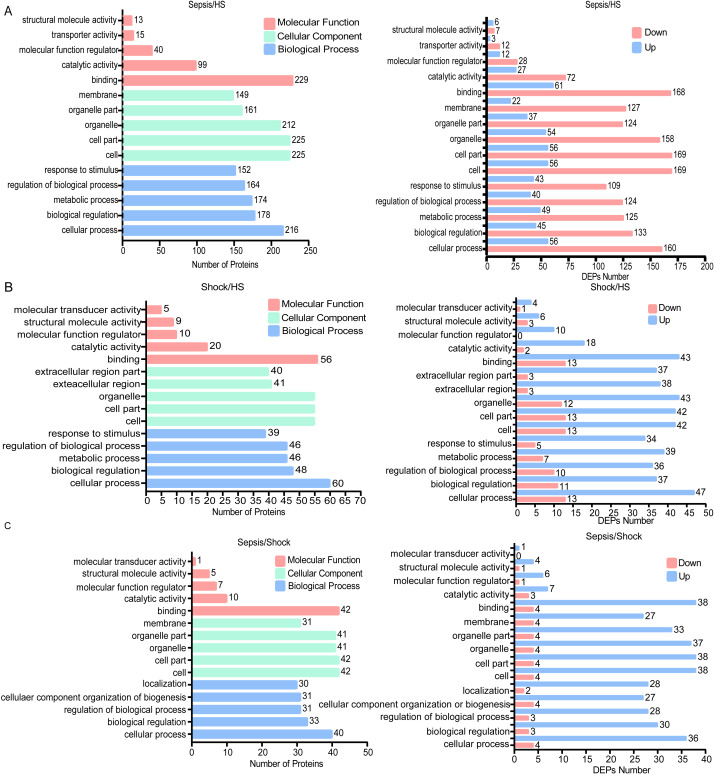
Top five functional GO enrichment analysis of identified proteins between all sepsis and HS. (A) Top five Functional GO Classification of all DEPs and up- and down-regulated DEPs between patients with sepsis and HS. (B) Top five Functional GO Classification of all DEPs and up- and down-regulated DEPs between patients with septic shock and HS. (C) Top five Functional GO Classification of all DEPs and up- and down-regulated DEPs between patients with sepsis and septic shock.

### GO enrichment analysis

The functional significance of all identified proteins was assessed through GO annotation analysis using Blast2GO software.

The top five functional GO enrichment analysis of DEPs were comparatively analyzed between sepsis and HS. Comparative analysis results revealed the most prevalent biological processes, including ‘Cellular processes’, ‘Biological regulation’ and ‘Metabolic process’, out of 26 processes. The most enriched cellular components, out of 16 components, were found to be ‘cell’, ‘cell part’, and ‘organelle’. In terms of molecular functions, the most abundant functions, out of 13 possibilities, were ‘binding’ and ‘catalytic activity’. Based on these findings, a GO function classification map was generated to depict these DEPs (Fig. 3A). This map was then utilized to discern the up- and down-regulated proteins (Fig. 3A). Additionally, when comparing patients with septic shock to HS, the results revealed that the most prevalent biological processes (out of 26) were ‘Cellular process’, ‘Biological regulation’, and ‘Metabolic process’. The most enriched cellular components (out of 16) were ‘Cells’, ‘Cell parts’ and ‘Organelles’. In terms of molecular functions (out of 13), the most common were ‘Binding’ and ‘Catalytic activity’. Based on these findings, we generated a functional classification map of GO to represent these DEPs (Fig. 3B). We used this classification map to distinguish between up-regulated and down-regulated proteins (Fig. 3B). Furthermore, we analyzed and compared patients with sepsis and septic shock. The findings revealed that the most predominant biological processes out of a total of 26 were ‘Cellular process’, ‘Biological regulation’ and ‘Regulation of biological process’. In terms of cellular components, the most abundant ones out of 16 were ‘cell’ and ‘cell part’. Additionally, the most prevalent molecular functions out of 13 were ‘binding’ and ‘catalytic activity’. Based on these results, a functional classification map was created to represent all DEPs, as depicted in Fig. 3C. The map was utilized to differentiate between up-regulated and down-regulated proteins, as illustrated in Fig. 3C.

We also performed GO analysis comparing all sepsis patients (both sepsis and septic shock combined) to HS. We obtained similar results: DEPs were enriched in biological processes such as ‘cellular process’ and ‘biological regulation’, cellular components such as ‘cell’, ‘cell part’ and ‘organelle’, and molecular functions such as ‘binding’ and ‘catalytic activity’. These differences were observed in both up- and down-regulated proteins (Fig. S3).

**Figure 4:**
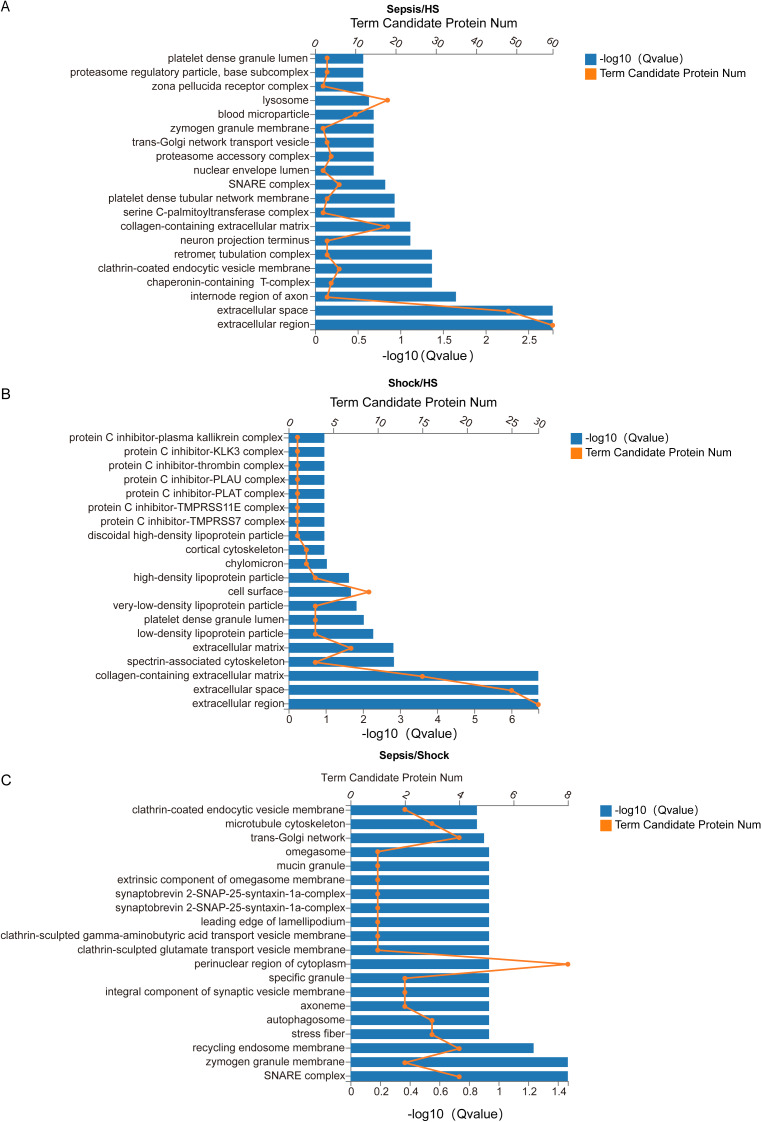
Histogram of GO enrichment for DEPs between all sepsis patients with HS. (A) Histogram of GO enrichment for DEPs between patients with sepsis and HS. The length of the lower bar on the *X*-axis represents the size of the (−log10 (Qvalue)) and the folded line on the upper *X*-axis represents the number of annotated differential proteins for each GO Term. (B) Histogram of GO enrichment for DEPs between patients with septic shock and HS. The length of the lower bar on the *X*-axis represents the size of the (−log10 (Qvalue)) and the folded line on the upper *X*-axis represents the number of annotated differential proteins for each GO Term. (C) Histogram of GO enrichment for DEPs between patients with sepsis and septic shock. The length of the lower bar on the *X*-axis represents the size of the (−log10 (Qvalue)) and the folded line on the upper *X*-axis represents the number of annotated differential proteins for each GO Term.

We conducted enrichment analysis of GO entries for proteins exhibiting significant differences (referred to as DEPs) to obtain the enriched significance *P*-value and FDR-corrected Q-value for each entry. We then generated bar charts to visualize the results. Firstly, we compared patients with sepsis with HS and observed the most significant differences in ‘extracellular region’ and ‘extracellular space’ (Fig. 4A). When comparing patients with septic shock with HS, significant differences were observed in ‘extracellular region’, ‘extracellular space’ and ‘collagen-containing extracellular matrix’ (Fig. 4B). In addition, a comparative analysis was conducted between patients with sepsis and septic shock. This analysis revealed the most significant differences in ‘SNARE complex’ and ‘zymogen granule membrane’ (Fig. 4C). Collectively, these findings indicate that the sepsis and septic shock groups exhibit distinct alterations in the GO analysis of DEPs in extracellular region, suggesting a shift associated with disease progression.

### KEGG pathway analysis

**Figure 5:**
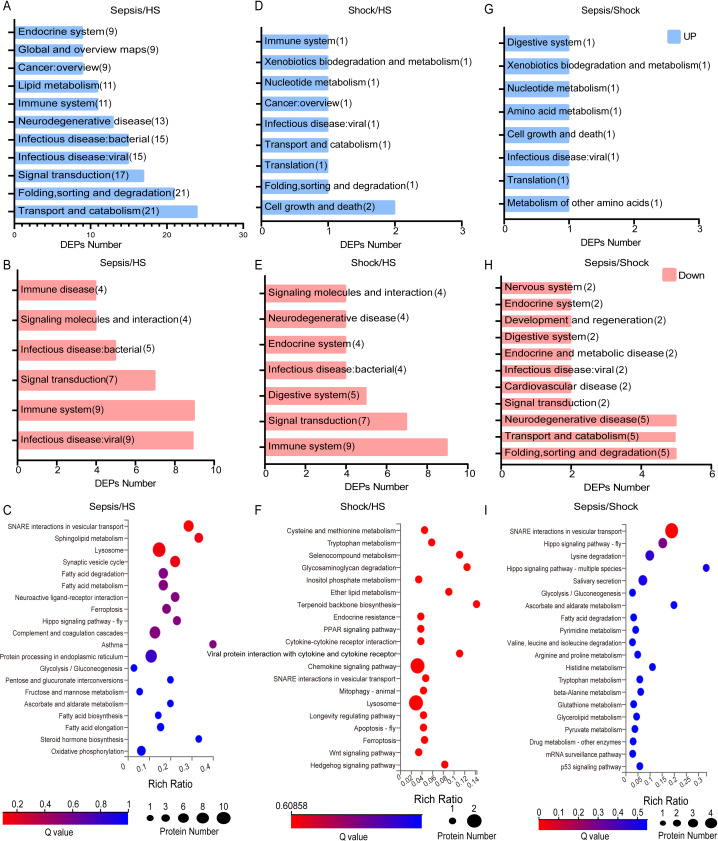
KEGG pathway classification of DEPs between all sepsis patients with HS. (A) Classification of KEGG pathways of up-regulated proteins in patients with sepsis and HS. (B) KEGG pathway classification of down-regulated proteins in patients with sepsis and HS. (C) KEGG Pathway enriched in DEPs between patients with sepsis and HS. (D) Classification of KEGG pathways of upregulated proteins in patients with septic shock and HS. (E) KEGG pathway classification of down-regulated proteins in patients with septic shock and HS. (F) KEGG Pathway enriched in DEPs between patients with septic shock and HS. (G) Classification of KEGG pathways of upregulated proteins in patients with sepsis and septic shock. (H) KEGG pathway classification of down-regulated proteins in patients with sepsis and septic shock. (I) KEGG Pathway enriched in DEPs between patients with sepsis and septic shock. The *x*-axis represents the enrichment ratio, which is calculated as the ratio of the number of proteins annotated to a pathway by a selected protein set to the number of proteins annotated to that pathway by the total protein set of the species. The *y*-axis corresponds to the KEGG Pathway, and the size of the bubbles indicates the number of proteins annotated to each pathway. Additionally, the color of the bubbles represents the significance value of the enrichment, with a redder color indicating a smaller significance value.

We conducted a KEGG pathway analysis, comparing patients with sepsis and HS, and observed that the up-regulated proteins were primarily enriched in 38 major pathways. Notably, the most significantly enriched pathways were ‘Transport and catabolism’, ‘Protein folding, sorting and degradation’, and ‘Signal transduction’ (Fig. 5A). In contrast, the down-regulated proteins were mainly enriched in ‘Infectious disease: viral’ and ‘Immune system’ (Fig. 5B). Figure 5C displays the 20 biological functions of the DEPs. Within the mentioned pathways, proteins such as CTSZ, TPP1, and ASAH1 were enriched in ‘Lysosome’. Additionally, our samples showed enrichment of metabolic and immune-related processes in the identified DEPs.

Similarly, in the septic shock *vs* HS comparison, upregulated DEPs were enriched in 35 pathways. The most significantly enriched pathway was ‘Cell growth and death’ (Fig. 5D). However, the down-regulated proteins were primarily associated with the ‘immune system’, ‘signal transduction’ and other pathways (Fig. 5E). Figure 5F displays 20 biological functions of DEPs. Furthermore, within the mentioned pathways, proteins like CTSZ and NAGLU were found to be enriched in ‘Lysosome’. At the same time, we found that proteins like PLCB3 and CXCL5 were found to be enriched in ‘Chemokine signaling pathway’. Additionally, some metabolic and immune-related processes were also enriched in the DEPs detected in our samples. In addition, a comparative analysis was performed between patients with sepsis and those with septic shock. The analysis revealed that the up-regulated proteins were primarily associated with 27 major pathways. Notably, the pathways of ‘Translation’ and ‘Metabolism of other amino acids’ were the most enriched (Fig. 5G). However, the down-regulated proteins were mainly enriched in pathways including ‘Folding, sorting and degradation’, ‘Transport and catabolism’, and ‘Neurodegenerative disease’ (Fig. 5H). Furthermore, Fig. 5I depicts 20 biological functions. Among the mentioned pathways, we observed that ‘SNARE interactions in vesicular transport’ showed enrichment in proteins such as VAMP8, VAMP2, STX16, and SNAP23. Furthermore, our data suggest the possibility of an enrichment of metabolic and immune-related processes among the DEPs, warranting a more in-depth analysis of the molecular mechanisms underlying these observations.

We also analyzed the differences between patients with different severity of sepsis with HS. Our analysis revealed enrichment of pathways related to ‘Infectious disease: viral’ and ‘Immune system’. Moreover, the results of this KEGG pathway analysis were shown in Fig. S4. Our results suggested a potentially significant correlation between the spectrum of sepsis severity and catabolism, indicating a need for further investigation into the metabolic alterations that accompany the progression of this systemic inflammatory response.

### Protein interaction network analysis

**Figure 6:**
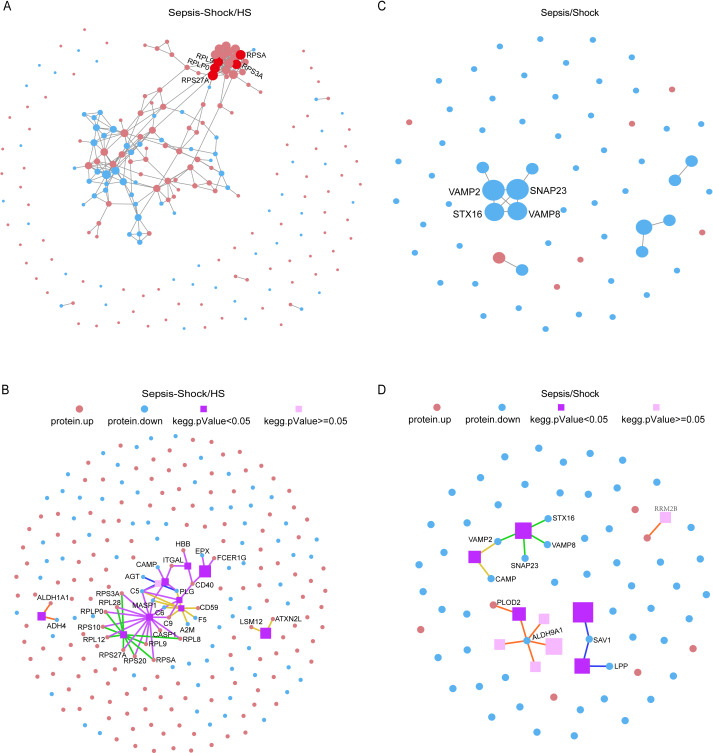
PPI network diagram. (A) PPI network diagram of DEPs between all sepsis patients and HS. Red indicates up-regulated proteins, and blue indicates down-regulated proteins. Circle size represents node degree. (B) PPI KEGG network diagram of DEPs between all sepsis patients and HS.Red and blue dots represent up-regulated and down-regulated DEPs respectively. Purple spheres denote the top 10 enriched pathways, with darker shades indicating statistically significant enrichment and lighter shades representing non-significant enrichment. The sphere size corresponds to the degree of enrichment (larger area indicates higher enrichment). Pathway categories are color-coded: red for Cellular Processes, blue for Environmental Information Processing, green for Genetic Information Processing, purple for Human Diseases (animal-specific), orange for Metabolism, yellow for Organismal Systems, and brown for Drug Development. Connecting lines between elements are similarly color-coded according to their respective pathway categories. (C) PPI network diagram of DEPs between patients with sepsis and septic shock. (D) PPI network diagram of DEPs between patients with sepsis and septic shock.

Next, we performed protein–protein interaction (PPI) network analysis on the 236 DEPs to identify functional modules (Fig. 6A). This analysis revealed proteins involved in protein translation, including RPLP0, RPL9, RPSA, and RPS3A, particularly within the ribosome. Further analysis demonstrated that RPS27A had the highest number of connected proteins, identifying it as the most central hub protein among all the DEPs. Meanwhile, we analyzed KEGG Pathways network between all sepsis patients and HS. PPI data showed that Human diseases may become a novel KEGG pathway to distinct all patients with sepsis and HS (Fig. 6B). Furthermore, we analyzed 65 DEPs between patients with sepsis and septic shock. The results showed that the proteins VAMP2, SNAP23, STX16, and VAMP8 might serve as pivotal biomarkers for differentiating between patients with sepsis and those with septic shock (Fig. 6C). Furthermore, we analyzed KEGG Pathways network between patients with sepsis and septic shock. PPI data also showed that Human diseases may become a novel KEGG pathway to distinguish patients with sepsis from those with septic shock (Fig. 6D).

Then, we analyzed the 316 significant DEPs in the previous screening with network interaction analysis and KEGG pathways network to examine their protein–protein relationships between patients with sepsis and HS (Fig. S5). Then, we analyzed the 83 significant DEPs in the previous screening with network interaction analysis and KEGG pathways network to examine their protein–protein relationships between patients with septic shock and HS (Fig. S6).

**Figure 7:**
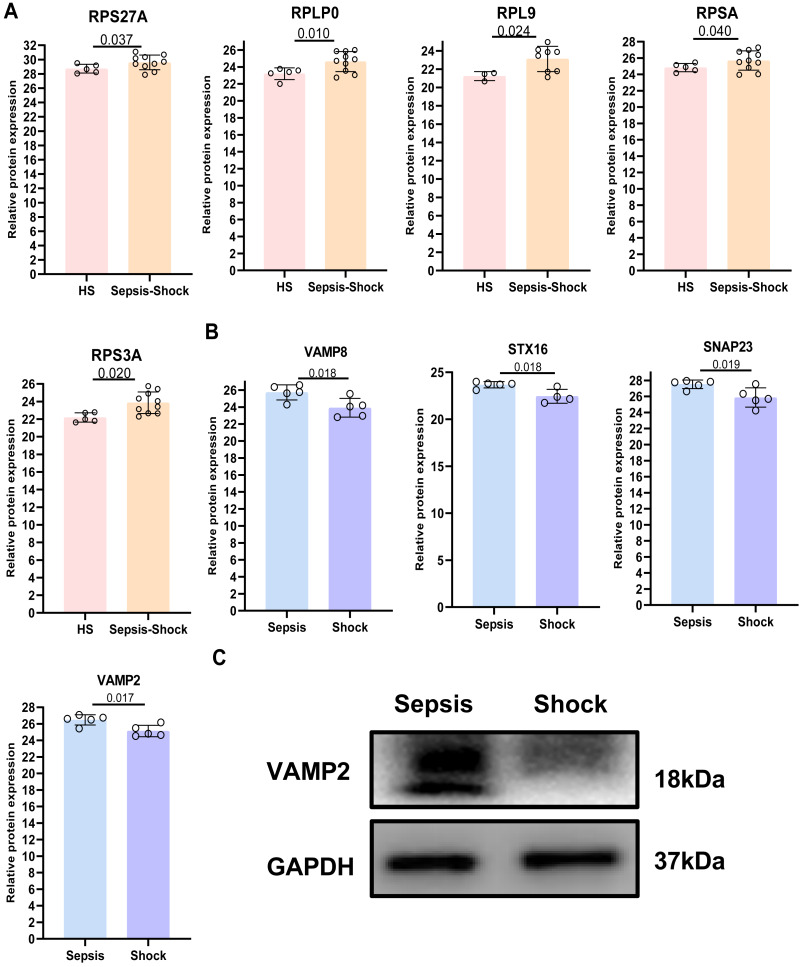
Relative protein expression among all sepsis patients and HS. (A) Relative protein expression of five DEPs among all sepsis patients and HS. (B) Relative protein expression of four DEPs between patients with sepsis and septic shock.(C) The expression of VAMP2 in human platelets was determined using western blot(*n* = 2). Abbreviations are as follows: M, maker; VAMP2, vesicle-associated membrane protein 2; GAPDH, glyceraldehyde -3-phosphate dehydrogenase.

Consistent with the results of PPI results, the relative protein expression of RPS27A, RPLP0, RPL9, RPSA, and RPS3A was significantly increased in platelets from all sepsis patients (sepsis and septic shock) compared with HS platelets (Fig. 7A). In addition, the relative protein expression of VAMP2, SNAP23, STX16, and VAMP8 was significantly decreased in platelets from septic shock compared with sepsis platelets (Fig. 7B). We have found that two proteins, VAMP2 and SNAP23, exhibited a negative correlation with prothrombin time (PT) (Figs. S7A–S7D). Among these, VAMP2 showed the most significant alteration based on our sequencing data (*p* = 0.017). Therefore, we selected VAMP2 for further validation *via* WB. Consistent with the proteomic findings, WB analysis confirmed that VAMP2 protein levels were significantly downregulated in the septic shock group compared to the sepsis group (Fig. 7C). The concordant downregulation strengthens the potential of VAMP2 as a differential biomarker between sepsis and septic shock. In parallel, we observed elevated P-selectin levels (Fig. S7E–S7F), indicating enhanced platelet activation in patients with sepsis.

## Discussion

Platelets, as the initial responders to dysregulated inflammatory responses, exhibit sensitivity to alterations in transcription and translation, leading to the synthesis of novel proteins at the onset of sepsis (Xu et al., 2024). Relevant previous studies have reported changes in the expression of mRNAs in sepsis patients and experimental models of sepsis, utilizing parallel methodologies such as RNA-sequencing (RNA-seq) and ribosome profiling (ribosome footprint profiling) (Middleton et al., 2019). Here, we performed a comparative analysis of platelet protein expression in patients with sepsis and septic shock using DIA-MS. Research has shown that early identification of high-risk patients and implementation of effective treatment can decrease mortality rates (Luo et al., 2021). Our novel findings may provide predictive biomarkers for the progression across various severity degrees of sepsis.

Currently, multiple lines of evidence indicate that protein expression plays a pivotal role in the pathogenesis of sepsis. Previous research has demonstrated that five proteins, including high-mobility group box 1 (HMGB1), matrix metalloproteinase-8 (MMP-8), neutrophil gelatinase-associated lipocalin (NGAL), lactotransferrin (LTF), and grancalcin, are robustly associated with septic patients, as identified through DIA analysis (Tong et al., 2019). Furthermore, an additional proteomics study has revealed the identification of five proteins with elevated expression levels in platelet samples from septic patients compared to healthy controls. These proteins include EF-hand calcium-binding domain-containing protein 7, actin, interleukin-1β, glycoprotein IX, and glycoprotein IIB (Liu, Li & Deng, 2014). However, the existing studies have primarily concentrated on comparing the outcomes of septic patients and HS, with a notable absence of research focusing on the variations in DEPs among patients with severity degrees of sepsis. Notably, we identified 50 DEPs among patients presenting with varying severity degrees of sepsis. Specifically, we outline that proteomic differences could arise through several mechanisms related to platelet count dynamics: (1) the bone marrow release of newly-formed platelets in response to thrombocytopenia, (2) altered platelet consumption pathways, and (3) pathogen infection of the platelet proteome by circulating factors such as LPS. We also cite the work of Middleton et al. (2019) and Middleton et al. (2019), which acknowledges that thrombocytopenia itself may impact on transcriptional and translational analyses in sepsis, supporting the relevance of this consideration.

PPI analysis revealed another major cluster, particularly within the ribosome (*e.g.*, RPS27A, RPSA, RPL9, RPS3A (Li, Li & Chen, 2023), and RPLP0 (Wang et al., 2019)). It has been documented that RPS3A can migrate to mitochondria to maintain brown adipocyte function in coronary artery disease (Tang et al., 2018). The release of inflammatory mediators, hypoxia, and reactive oxygen species (ROS) caused by sepsis directly damages mitochondria and trigger apoptosis or necrosis. Studies have shown that RPS27A can regulate cell apoptosis and proliferation in lung adenocarcinoma cells (Li et al., 2022a). RPS27A can also act as an inflammatory gene, causing inflammation and immune regulation disorders in various diseases (such as cerebral ischemia-reperfusion injury, diabetic retinopathy, and periodontitis), and accelerating the M2 polarization of macrophages (Huang & Zhou, 2022; Jinagal et al., 2024; Kuai et al., 2024; Li et al., 2024; Ma et al., 2019). Moreover, studies have demonstrated that RPLP0 can inhibit cell growth and induce cell apoptosis (Chang & Xu, 2022; Wang et al., 2019). It has been demonstrated that RPL9 is present in the serum of lipopolysaccharide (LPS)-stimulated septic mice and plays a regulatory role in its inflammatory response (Watanabe et al., 2022). Its pro-inflammatory effect has been confirmed in many studies and can cause apoptosis (Jian et al., 2024; Wang et al., 2025a; Watanabe et al., 2024). RPL9 has also been reported to activate the innate immune response (Huang et al., 2020). Consistent with our results, another gene network analysis of sepsis also found that RPL9 may be a key gene for changes in pediatric sepsis (Yang, Zhang & Wang, 2016). RPSA has recently been identified as a nuclear protein that recognizes viral nucleic acids and can promote the expression of pro-inflammatory cytokine genes in antiviral innate immunity (Jiang et al., 2023). More importantly, RPSA undergoes different changes after infection by various pathogens, confirming its mediation of multiple infectious diseases (Liu et al., 2021). Thus, we suppose that RPS27A, RPLP0, RPL9, RPSA and RPS3A may be used as new proteins to distinguish pediatric sepsis and HS.

Furthermore, according to PPI results, we found changes in the levels of four key proteins (VAMP2, VAMP8, STX16, and SNAP23) in patients with septic shock compared to those with sepsis. VAMP2 mainly participates in the development of sepsis by regulating immune responses, the release of inflammatory mediators, and endothelial barrier dysfunction. Studies have revealed that VAMP2 shows a significant positive correlation with immune infiltration (Chen et al., 2025; Liu et al., 2022). Besides, a previous study demonstrated that VAMP-2 is involved in the MR REDOX signaling pathway and forms SNARE complexs to promote endothelial dysfunction (Han et al., 2011). Inhibition of VAMP-2 expression reduces the secretion of cyclin A (CyPA), a factor known to promote inflammation, VSMC growth and endothelial cell apoptosis (Suzuki et al., 2006). VAMP-8 is a protein that mediates the fusion of autophagosomes with lysosomes, one of the key processes in sepsis (Li et al., 2023; Xia et al., 2019). VAMP8 has been reported to be essential for chemical and infectious colitis (Cornick et al., 2019) as well as viral infections (Cheng et al., 2019; Van Tol et al., 2020). Moreover, studies have suggested VAMP8 as a biomarker and potential therapeutic target for endothelial dysfunction in atherosclerosis (Yang et al., 2025). VAMP8 can also drive inflammation and tissue destruction by secreting mucin and interleukins through exocytosis (Cornick et al., 2017; Rosenfeld et al., 2025). STX16 also regulates lysosomal and autophagolysosomal biogenesis, and knockdown of STX16 leads to defects in lysosomal biogenesis (Gu et al., 2019). In other pathogen infections, the expression of STX16 and SNAP23 was inhibited (Zhang et al., 2004). SNAP23 can form complexes to regulate endothelial exocytosis and thus regulate vascular thrombosis and inflammation (Zhu, Yamakuchi & Lowenstein, 2015). As a component of the cellular mechanism required for the fusion of intracellular transport vesicles with target membranes, SNAP23 plays a key role in inflammation, immunity, and autophagy by promoting degranulation through its phosphorylation (Chariot, 2009; Chen et al., 2022; Feng et al., 2018; Li et al., 2022b; Schoppmeyer et al., 2022; Shariq et al., 2023). Reports have shown that TAT-SNAP23 treatment inhibits the priming of neutrophil functions contributing to shock and/or sepsis-induced extrapulmonary acute lung injury (Bai et al., 2015). The above evidence suggests that these four proteins could potentially distinguish between septic shock and sepsis.

Key limitations of this study are as follows. First, the sample size (*n* = 15) may limit the generalizability of the findings. Second, we assessed only platelet activation (P-selectin) and coagulation parameters; aggregation, apoptosis, and necrosis were not evaluated. Despite these limitations, our data provide preliminary insight into sepsis-related alterations in the platelet proteome. Studies with larger cohorts and broader functional profiling are warranted.

## Conclusions

In conclusion, our preliminary finding identifies proteomic changes that may distinguish sepsis severity and potentially serve as candidate biomarkers. These results, however, are constrained by the small sample size and the limited functional assessments, and must be validated in future larger-scale studies.

## Supplemental Information

10.7717/peerj.20844/supp-1 Supplemental Information 1 Demographic data for the cohort of septic patients and healthy subjects

10.7717/peerj.20844/supp-2 Supplemental Information 2 Number of up- and down-regulated DEPs (A) c between sepsis and HS, septic shock and HS and sepsis and septic shock. (B) Number of up- and down-regulated DEPs between all sepsis and HS.

10.7717/peerj.20844/supp-3 Supplemental Information 3 F unctional GO classification of up- and down-regulated DEPs between all patients with sepsis and HS

10.7717/peerj.20844/supp-4 Supplemental Information 4 KEGG pathway enrichment of up- and down-regulated DEPs between all patients with sepsis and HS

10.7717/peerj.20844/supp-5 Supplemental Information 5 PPI network diagram (A) PPI network diagram of DEPs between sepsis patients and HS. (B) PPI KEGG network diagram of DEPs between sepsis patients and HS.

10.7717/peerj.20844/supp-6 Supplemental Information 6 PPI network diagram (A) PPI network diagram of DEPs between patients with septic shock and HS. (B) PPI KEGG network diagram of DEPs between patients with septic shock and HS.

10.7717/peerj.20844/supp-7 Supplemental Information 7 Supplementary Figure 7: Functional assessment of platelets (A) Results of the correlation analysis between VAMP2 expression levels and prothrombin time (PT). (B) Results of the correlation analysis between SNAP23 expression levels and prothrombin time (PT). (C) Results of the correlation analysis between STX16 expression levels and prothrombin time (PT). (D) Results of the correlation analysis between VAMP8 expression levels and prothrombin time (PT). (E) Representative flow cytometry plot of P-selectin. (F) Statistical results of P-selectin.

10.7717/peerj.20844/supp-8 Supplemental Information 8 Raw data for Fig. 7

10.7717/peerj.20844/supp-9 Supplemental Information 9 Proteomics data of 15 samples (236 DEPs)

10.7717/peerj.20844/supp-10 Supplemental Information 10 Raw data for Table 1

10.7717/peerj.20844/supp-11 Supplemental Information 11 Proteomics data of 15 samples (32 DEPs)

10.7717/peerj.20844/supp-12 Supplemental Information 12 Raw WB data for VAMP2 and GADPH

## Abbreviations

DIA-MS: data independent acquisition mass spectrometry
HS: healthy subjects
DEPs: differentially expressed proteins
KEGG: Kyoto Encyclopedia of Genes and Genomes
GO: Gene Ontology
PPI: protein–protein interaction
mRNAs: messenger RNAs
RPS27A: 40S ribosomal protein S27a
RPLP0: ribosomal protein large P0
RPL9: 60S ribosomal protein L9
RPSA: Ribosomal Protein SA
RPS3A: ribosomal protein S3a
VAMP: vesicle-associated membrane protein
STX16: syntaxin-16
SNAP23: synaptosomal-Associated Protein 23
DDA: data-dependent acquisition
DIA: data-independent acquisition
LPL: lipoprotein lipase
MIT: maximal injection time
minutes: min
FITC: fluorescein isothiocyanate
PS: phosphatidylserine
TMRM: tetramethylrhodamine methyl ester
DTT: dithiothreitol
IAM: iodoacetamide
BSA: bovine serum albumin
HS: healthy subject
ACN: acetonitrile

## Additional Information and Declarations

### Data Availability

The following information was supplied regarding data availability: The raw measurements are available in the Supplementary Files. The mass spectrometry proteomics data are available in the ProteomeXchange Consortium via the iProX partner repository: PXD053408/IPX0009129002.
