# Supplementary figures and images for: Platelet proteome for predictive diagnosis and differentiation of sepsis and septic shock in pediatric patients
Source: PeerJ. 2026 Mar 6;14:e20844. doi: 10.7717/peerj.20844 (PMC12970313; doi:10.7717/peerj.20844)

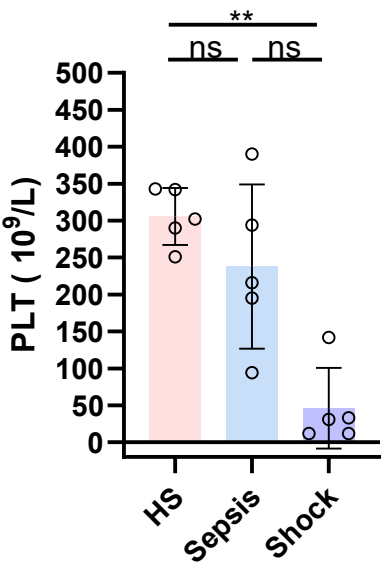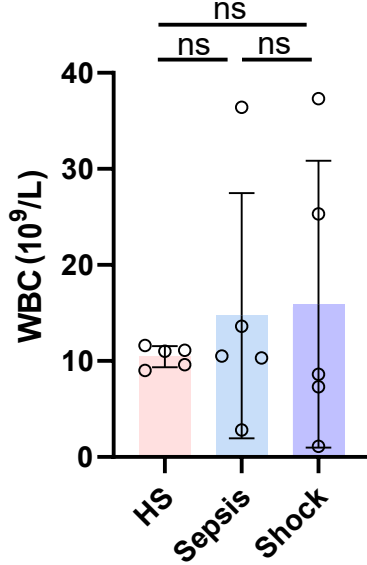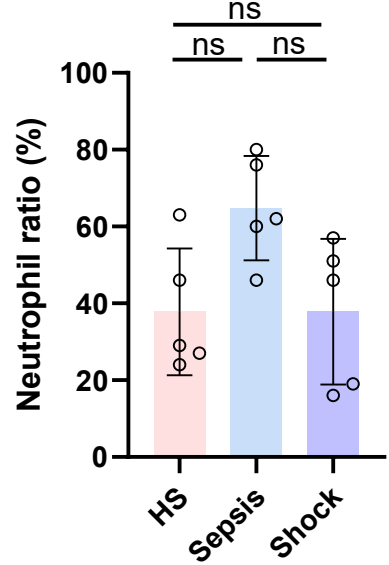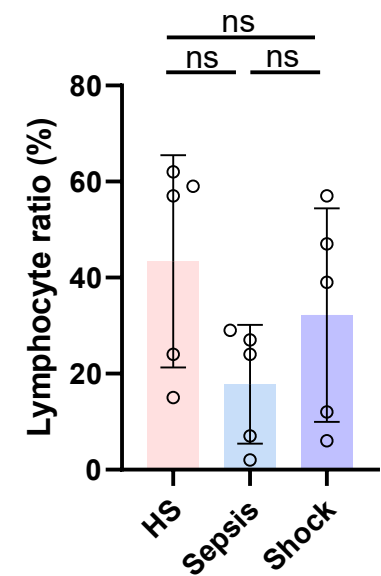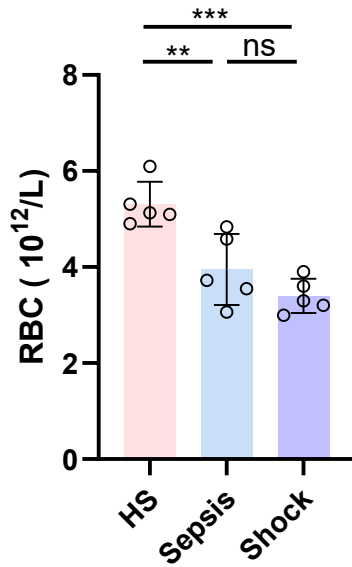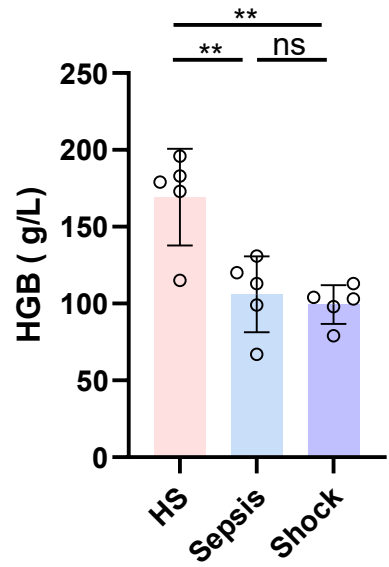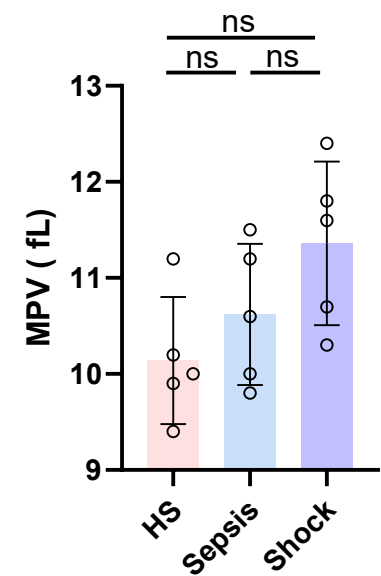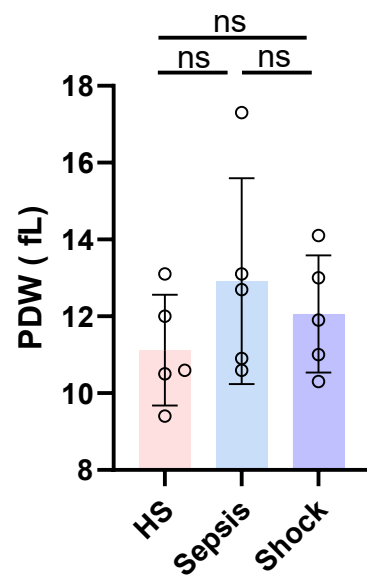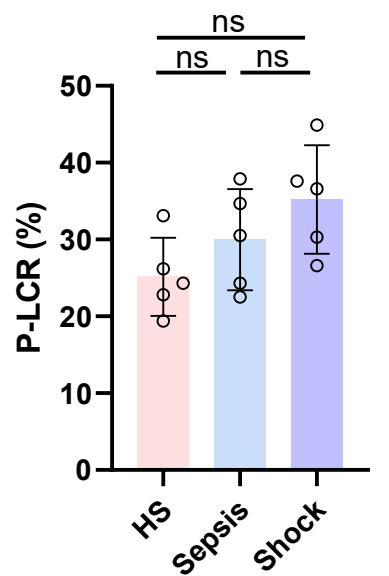

Supplement: Supplemental Information 1 [file peerj-14-20844-s001.pdf]

A

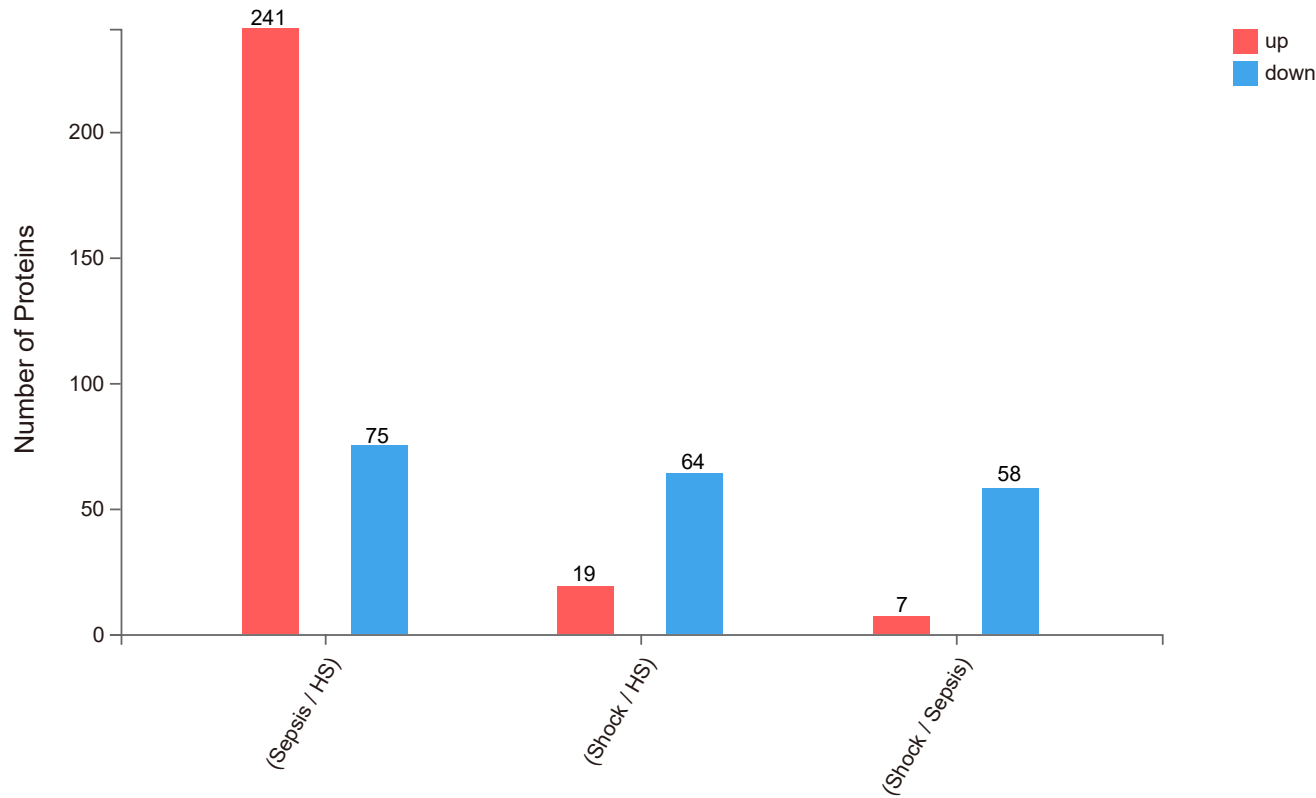

B

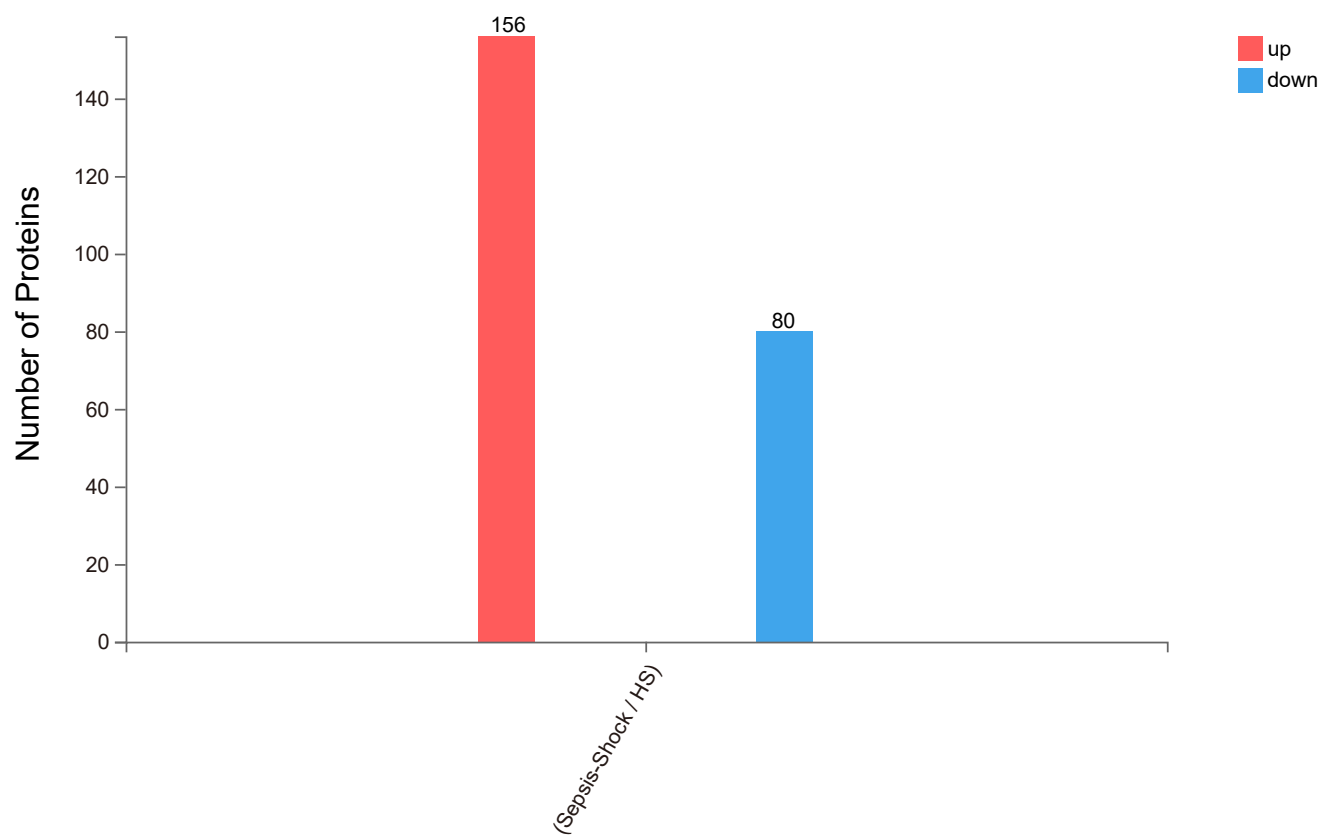

Supplement: Supplemental Information 2 — (A) c between sepsis and HS, septic shock and HS and sepsis and septic shock. (B) Number of up- and down-regulated DEPs between all sepsis and HS. [file peerj-14-20844-s002.pdf]

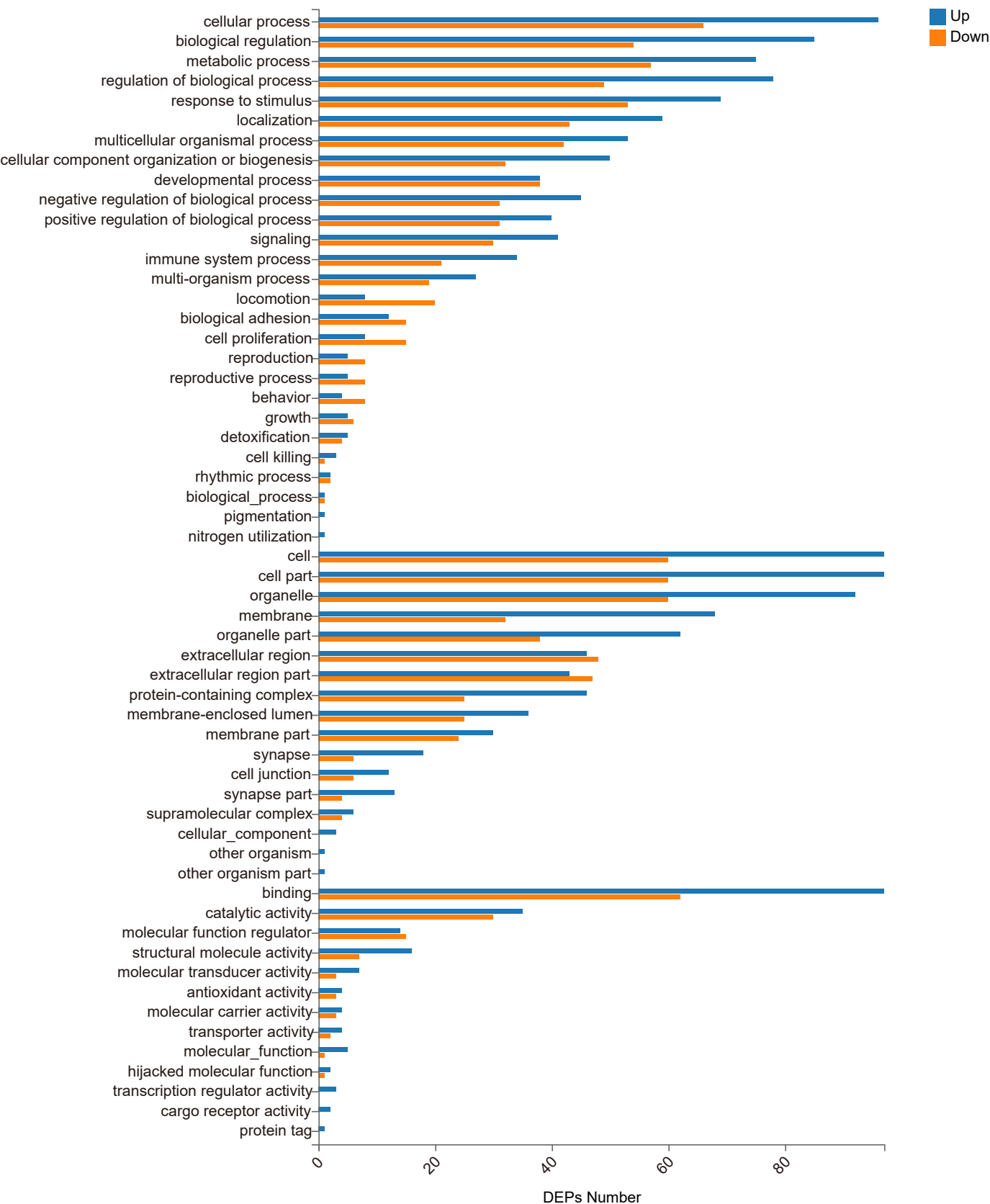

Supplement: Supplemental Information 3 [file peerj-14-20844-s003.pdf]

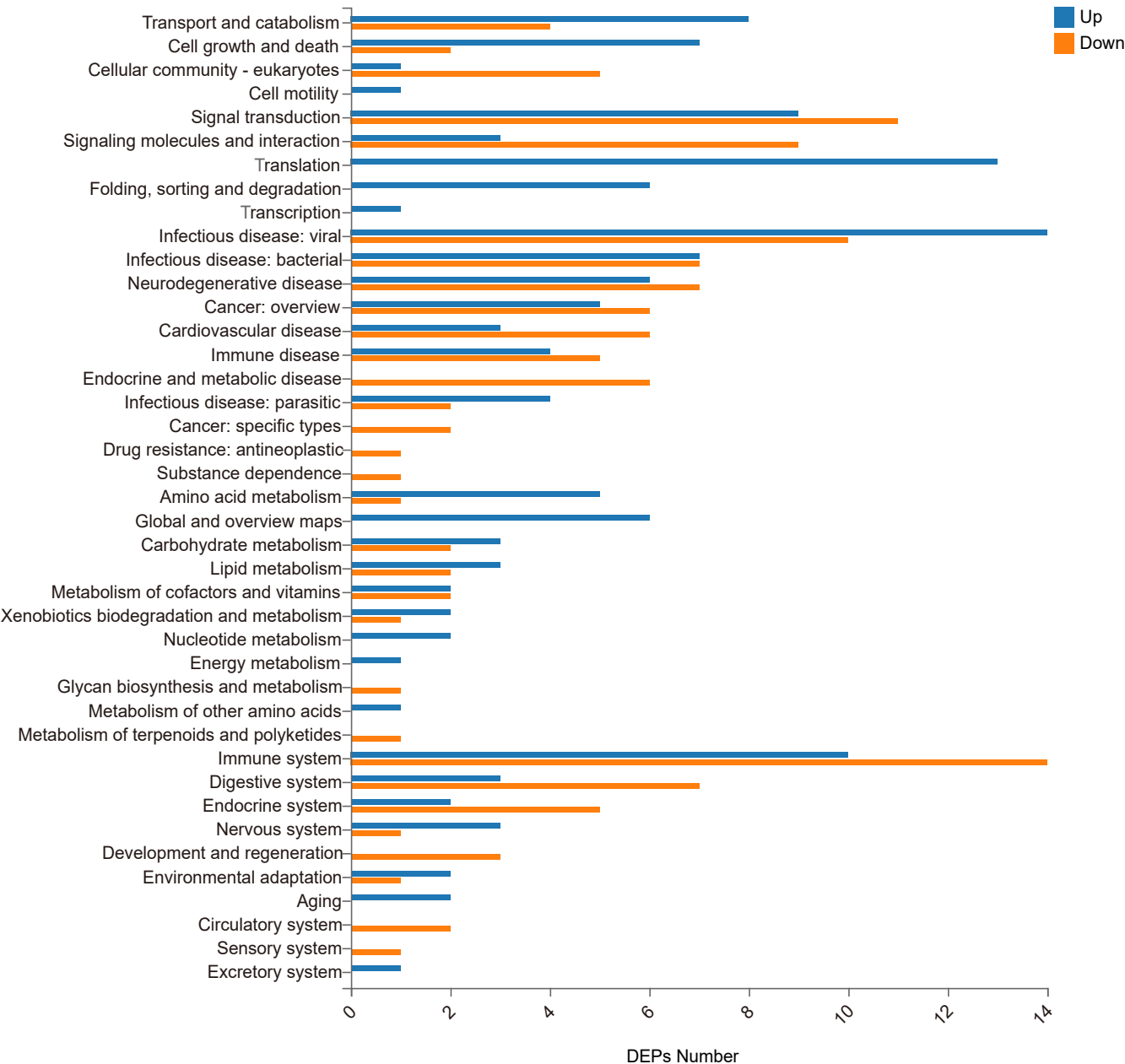

Supplement: Supplemental Information 4 [file peerj-14-20844-s004.pdf]

A

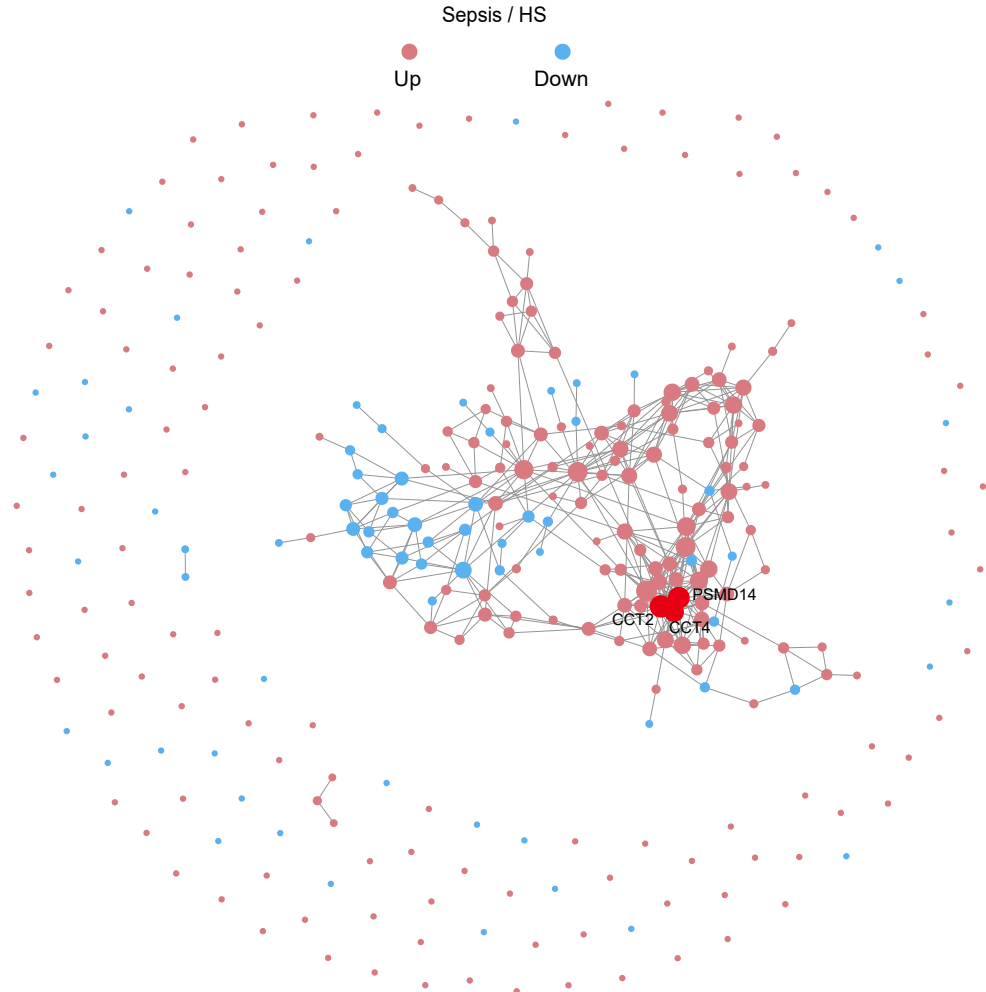

B

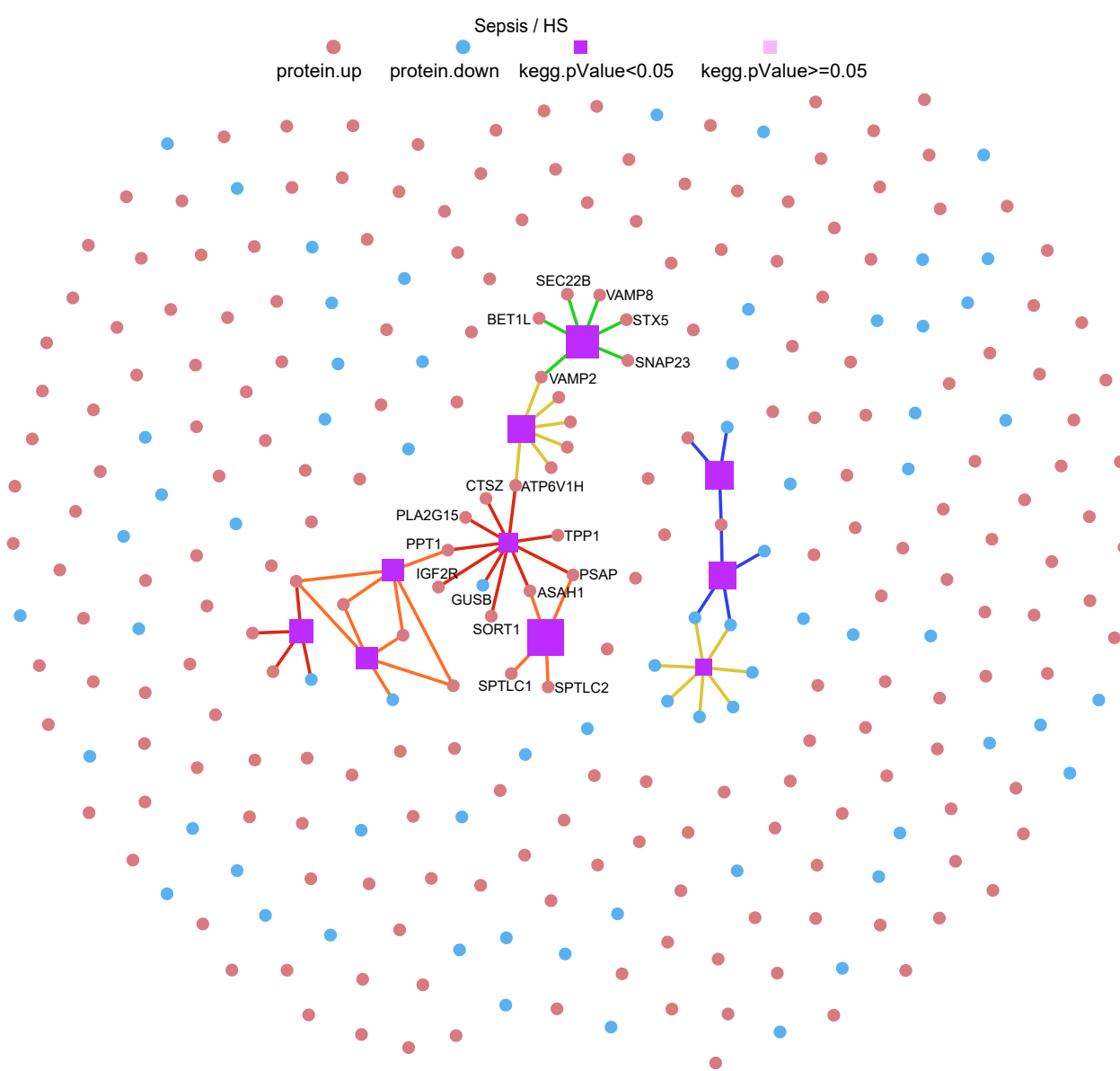

Supplement: Supplemental Information 5 — (A) PPI network diagram of DEPs between sepsis patients and HS. (B) PPI KEGG network diagram of DEPs between sepsis patients and HS. [file peerj-14-20844-s005.pdf]

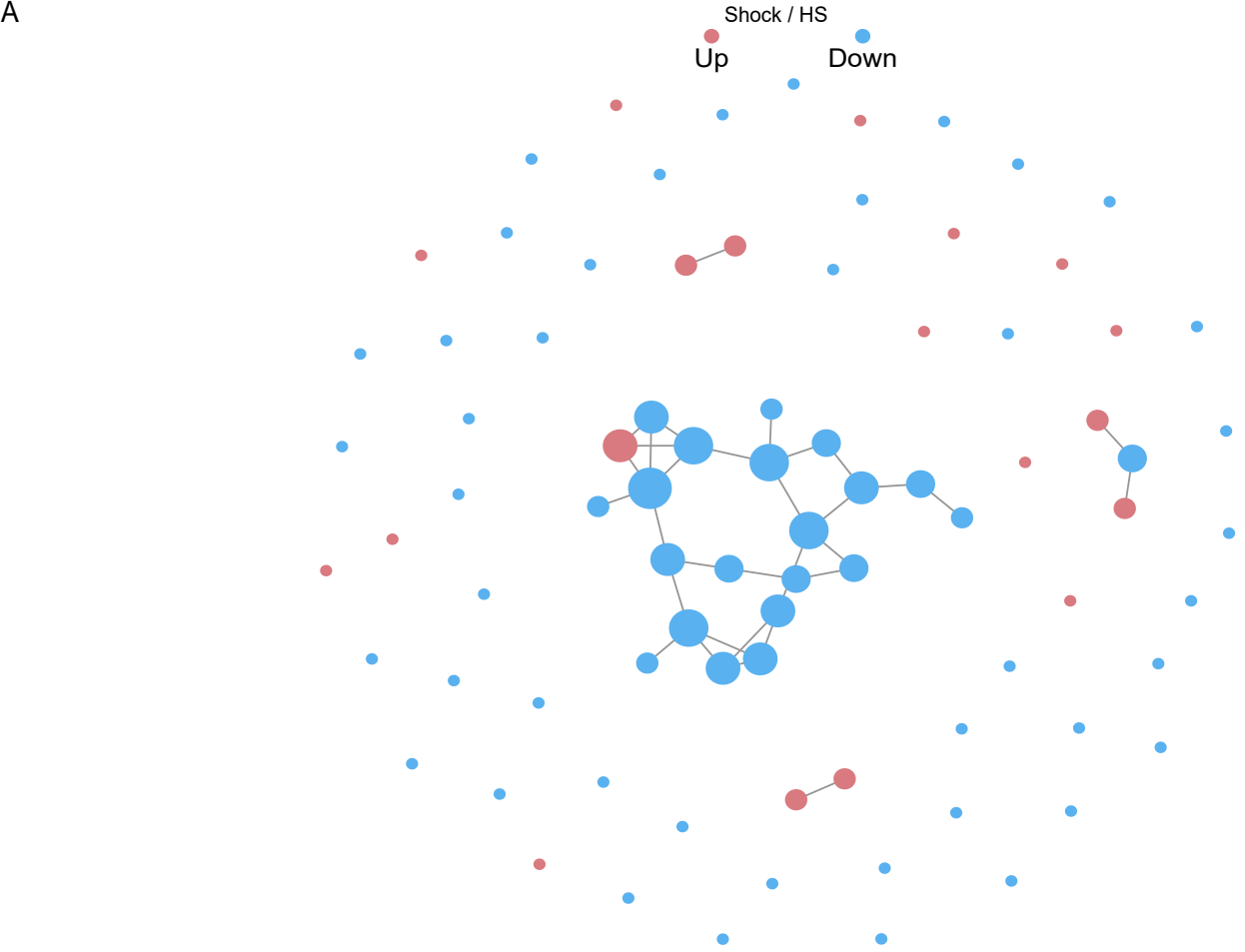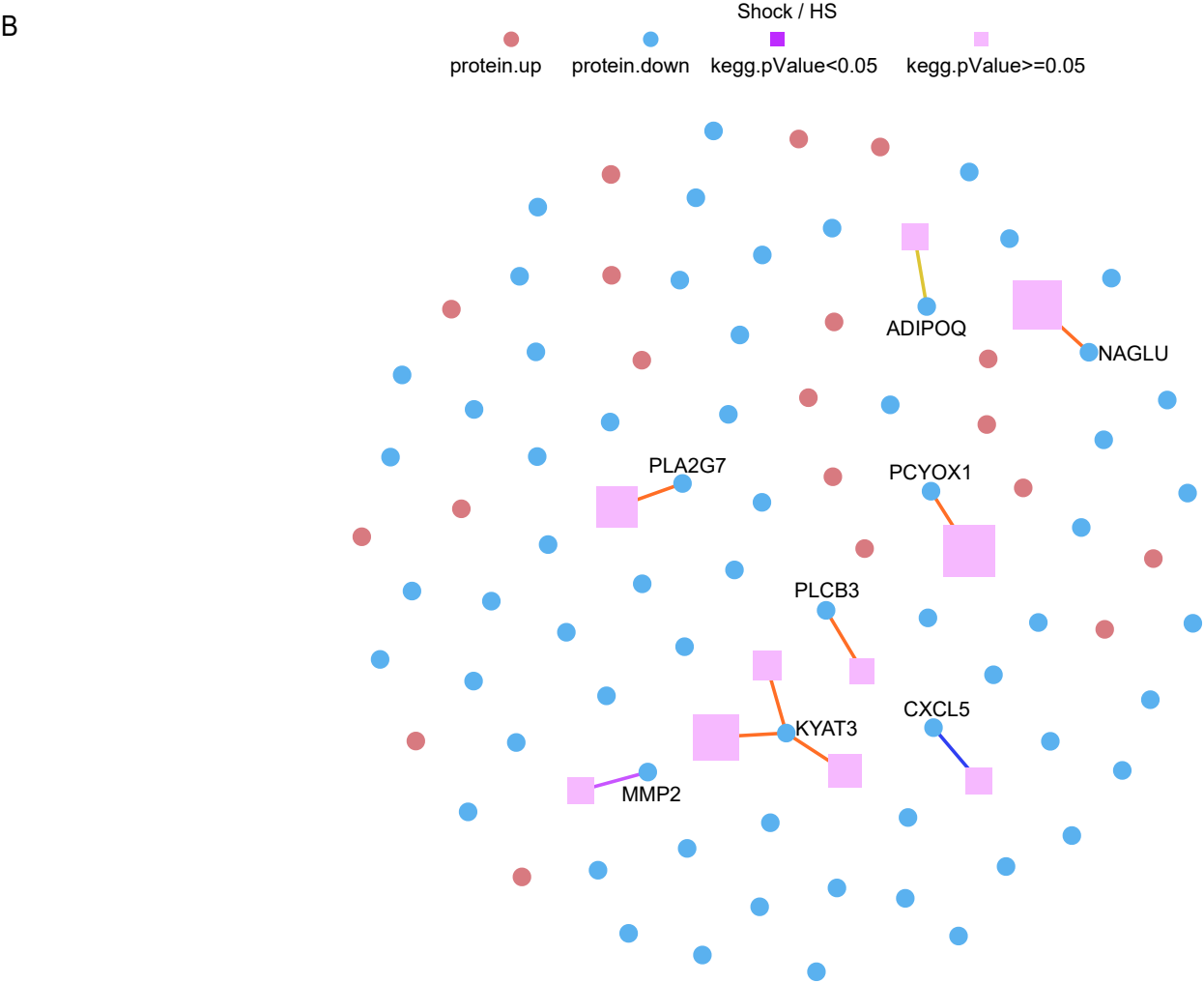

Supplement: Supplemental Information 6 — (A) PPI network diagram of DEPs between patients with septic shock and HS. (B) PPI KEGG network diagram of DEPs between patients with septic shock and HS. [file peerj-14-20844-s006.pdf]

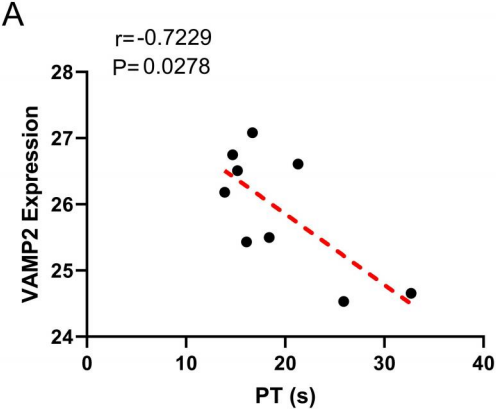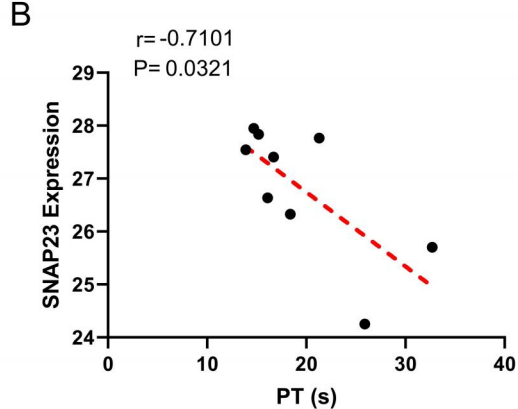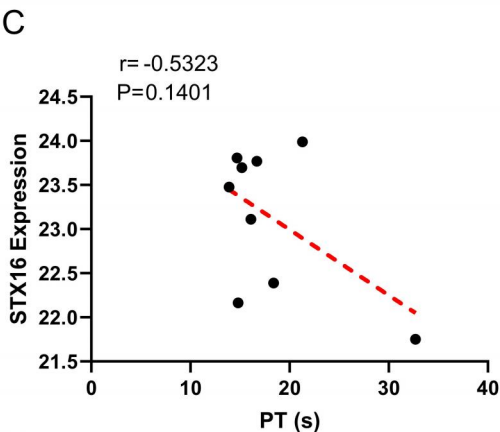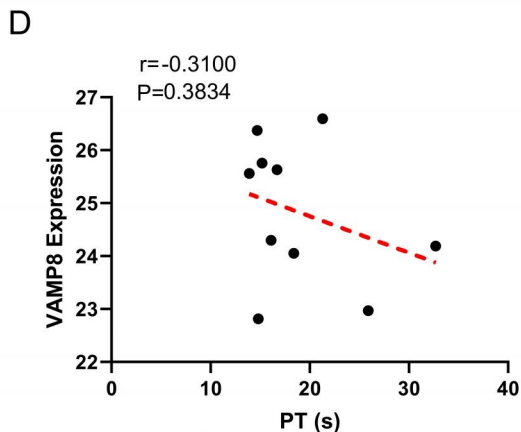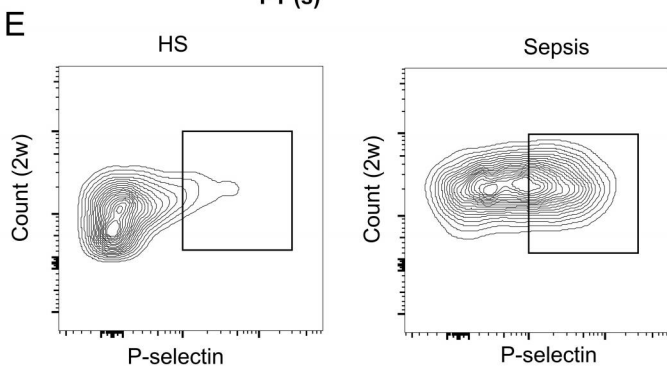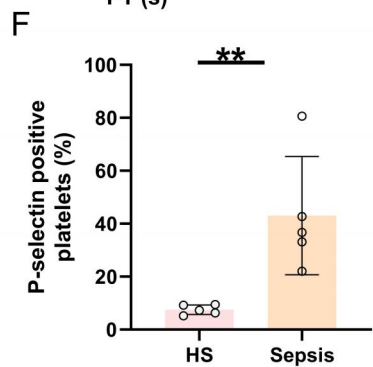

Supplement: Supplemental Information 7 — (A) Results of the correlation analysis between VAMP2 expression levels and prothrombin time (PT). (B) Results of the correlation analysis between SNAP23 expression levels and prothrombin time (PT). (C) Results of the correlation analysis between STX16 expression levels and prothrombin time (PT). (D) Results of the correlation analysis between VAMP8 expression levels and prothrombin time (PT). (E) Representative flow cytometry plot of P-selectin. (F) Statistical results of P-selectin. [file peerj-14-20844-s007.pdf]

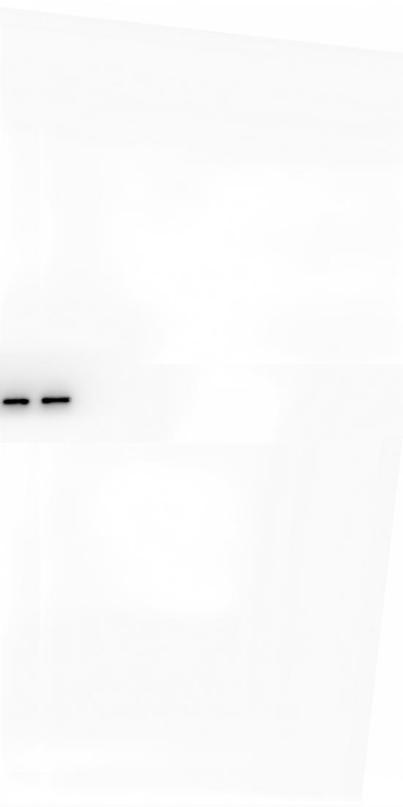


GADPH raw data


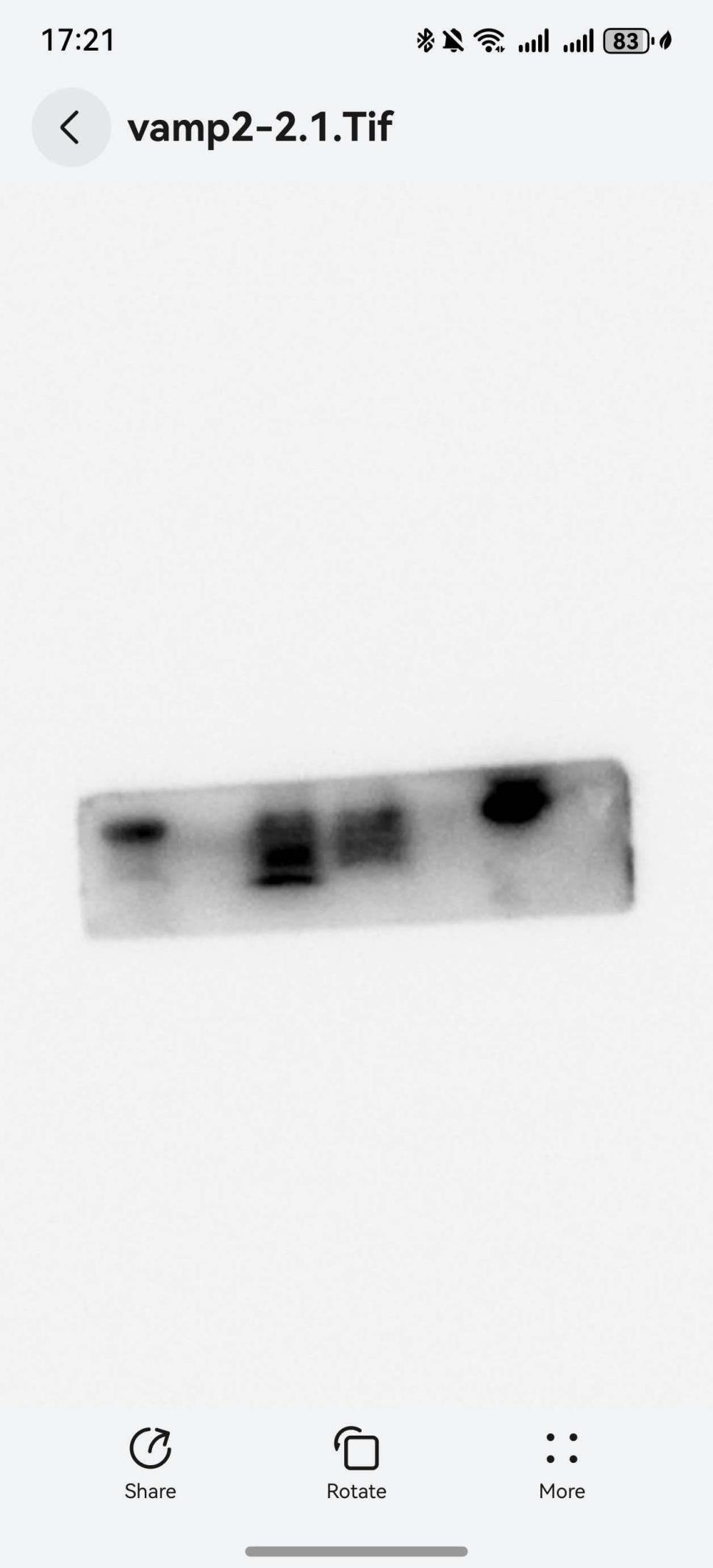


VAMP2 raw data

Supplement: Supplemental Information 12 [file peerj-14-20844-s012.docx]
